# Gene-gene and gene-environment interactions influence platinum-based chemotherapy response and toxicity in non-small cell lung cancer patients

**DOI:** 10.1038/s41598-017-05246-8

**Published:** 2017-07-11

**Authors:** Jia-Jia Cui, Lei-Yun Wang, Tao Zhu, Wei-Jing Gong, Hong-Hao Zhou, Zhao-Qian Liu, Ji-Ye Yin

**Affiliations:** Department of Clinical Pharmacology, Xiangya Hospital, Central South University, Changsha 410008; P. R. China; Institute of Clinical Pharmacology, Central South University; Hunan Key Laboratory of Pharmacogenetics, Changsha, 410078 P. R. China

## Abstract

Platinum-based chemotherapy is a major therapeutic regimen of lung cancer. Various single nucleotide polymorphisms (SNPs) reported were associated with platinum-based chemotherapy response and drug toxicity. However, neither of the studies explored this association from SNP-SNP interaction perspective nor taking into effects of SNP-environment consideration simultaneously. We genotyped 504 polymorphisms and explore the association of gene-gene and gene-environment interactions with platinum-based chemotherapy response and toxicity in 490 NSCLC patients. 16 SNPs were found significantly associated with platinum-based chemotherapy, and they were picked out as study object in the validation cohort. We recruited 788 patients in the validation cohort. We found that HSPD1 rs17730989-SUMF1 rs2633851 interaction was associated with platinum-based chemotherapy-induced hematologic toxicity (adjusted OR = 0.233, P = 0.018). In addition, the combined effect of ABCG2 rs2231142-CES5A rs3859104 was significantly associated with overall toxicity (adjusted OR = 8.044, P = 4.350 × 10^−5^). Besides, the model of ARHGAP26 rs3776332-ERCC6 rs2228528-SLC2A1 rs4658-histology was associated with platinum-based chemotherapeutic response. Gene-gene and gene-environment interactions have been identified to contribute to chemotherapy sensitivity and toxicity. They can potentially predict drug response and toxicity of platinum-based chemotherapy in NSCLC patients.

## Introduction

Lung cancer is one of the leading causes of cancer related death worldwide^1^. In China, lung cancer accounts for 17.09% and 21.68% cases of all cancer incidences and mortalities respectively^2, 3^. Lung cancer consists of non-small cell lung cancer (NSCLC) and small cell lung cancer (SCLC) in histology according to World Health Organization (WHO) classification, and NSCLC accounts for approximately 85% of all cases^4^. Due to the difficulty of early diagnosis, a great number of patients are diagnosed at advanced stages (III/IV) when surgery is no longer suitable for them. Thus, chemotherapy is a major treatment choice. Platinum is the first line treatment regimen for NSCLC, unfortunately, drug response and adverse drug reaction (ADR) varies largely among patients.

Our and others’ previous studies showed that genetic variations were one of the major causes for inter-individual difference of these phenotypes^5–9^. In the case of platinum-based chemotherapy, a number of SNPs have been reported to be associated with drug response as well as ADR^10, 11^. Previously, the association of genetic polymorphisms with complex quantitative traits was tested by univariate method. However, the SNPs identified so far can explain the phenotype traits far from satisfactory. Low power to detect gene-gene interaction can partly explain the “missing heritability”, thus further efforts other than the single-SNP analysis is needed^12^. Since analyzing one single SNP at a time is under powered for complex phenotypes of drug resistance and toxicity, we wanted to explore whether environmental factors combined with genes can achieve better predictive outcomes. In other words, we tried to improve the prediction accuracy by integrating multiple SNPs and clinical factors simultaneously in this manuscript, and results suggested that gene-gene and gene-environment interactions might influence these phenotypes^13^. In addition to our study, others also showed that interaction of SNPs and their synergistic effects could better explain inter-individual difference from the pharmacogenetics aspect. And hence it is becoming the focus to explore the association of genetic and clinical factors with complex phenotypes^14, 15^.

However, current pharmacogenomics studies of platinum-based chemotherapy in NSCLC only considered the effect of a single SNP and paid no attention to effects of gene-gene and gene-environment interactions. In our previous study, we analyzed 416 SNPs of 185 genes with platinum response and toxicity^13^. In the present investigation, based on these SNPs, we further explored the effects of gene-gene and gene-environment interactions on platinum-based chemotherapy response and toxicity in Chinese NSCLC patients.

## Results

### Clinical characteristics of subjects

490 subjects who had enough clinical data for sensitivity and toxicity evaluation were included in the discovery stage. Clinical data were collected and were classified by susceptibility, hematologic toxicity, gastrointestinal toxicity and overall toxicity. Details were presented in Supplementary Table S2A. All patients received at least two cycles of platinum-based chemotherapy. Among them, the response was evaluated in 328 patients, 111 (33.84%) subjects were responders and 217 (66.16%) individuals were non-responders. The toxicity was evaluated in all patients, 85 (17.35%) patients suffered severe gastrointestinal toxicity and 116 (23.67%) patients suffered hematologic toxicity.

To test the credibility of the discovery stage, we further investigated the SNPs in the validation cohort which enrolled 788 subjects. The clinical information was presented in Supplementary Table S2B. 781 subjects with sufficient susceptibility data were collected for drug response evaluation, and 788 subjects for gastrointestinal toxicity and 782 subjects for hematologic toxicity evaluation. As shown in Supplementary Table S2B, 151 (19.33%) subjects were responders, while 630 (80.67%) individuals were non-responders. Among them, 175 (22.21%) suffered severe hematologic toxicity and 66 (8.38%) patients suffered gastrointestinal toxicity. The clinical characteristics of all subjects were summarized in Table 1.

**Table 1:** Characteristics of all patients enrolled in this study.

Characteristics	Response		p	Overall toxicity		p	Hematological toxicity		p	Gastrointestinal toxicity		p
	Responders	Non-responders		Low toxicity	High toxicity		Low toxicity	High toxicity		Low toxicity	High toxicity	
Total	262	847		886	392		981	291		1127	151	
Gender			0.308			0.011			0.147			0.074
Male	195	603		660	265		720	201		836	89	
Female	67	244		226	127		261	90		291	62	
Age(years)			0.166			0.891			0.378			0.060
<60	142	500		523	233		588	166		656	100	
≥60	120	347		363	159		393	125		471	51	
Smoke condition			0.488			0.287			0.566			0.051
Smoker	142	500		366	175		411	128		466	75	
Non-smoker	120	347		520	217		570	163		661	76	
Histology			0.051			0.039			0.002			0.236
Adenocarcinoma	89	247		307	112		344	73		363	56	
Squamous cell	132	498		478	231		524	181		631	78	
Other	41	102		101	49		113	37		133	17	
Pathological stage			0.087			0.268			0.232			0.402
III	79	222		249	102		275	75		314	37	
IV	155	572		563	269		623	204		730	102	
Other	28	53		74	21		83	12		83	12	
PS												
1	144	582	0.000	530	206	0.032	567	164	0.468	671	65	0.001
2	103	247		309	158		353	113		398	69	
Other	15	18		47	28		61	14		58	17	

### Influence of gene-gene and gene-environment interaction to platinum-based chemotherapeutic response

**Figure 1:**
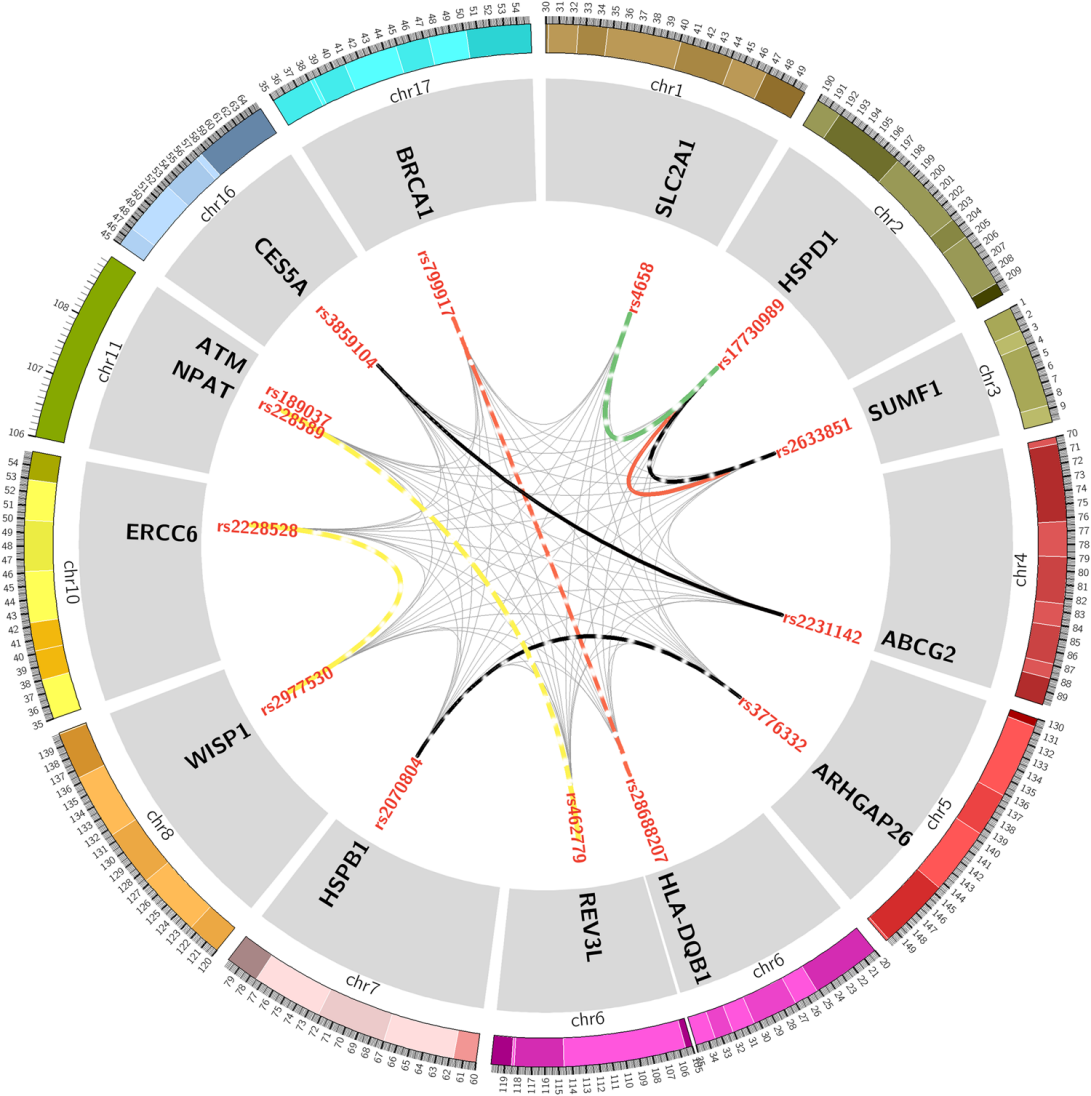
Paired gene-gene interaction in discovery stage, validation stage respectively. The chromosomes are arranged end by end in a clockwise direction. The exterior of the circle is chromosome number and scale. The inner circle represents the genes of SNPs located. The innermost circle is the name of SNPs. The lines connecting two SNPs represent paired gene-gene interactions. The thinner grey lines represent interactions with no statistical significance while thicker dotted line represent interactions with statistical significance in discovery stage and thicker solid line represent interactions replicated in the validation stage. Black lines represent overall toxicity, red lines hematologic toxicity, green line sensitivity and yellow lines gastrointestinal toxicity.

We first explored the paired gene-gene interaction to chemotherapy response. As mentioned above, we enrolled 504 SNPs in the first stage, a total of 16 interactional SNPs were found remarkably associated with chemotherapy response or toxicities in this cohort (Fig. 1 and supplementary Table S3) We found that the paired interaction between SLC2A1 rs4658 and HSPD1 rs17730989 was significantly associated with platinum-based chemotherapy response in NSCLC patients (adjusted OR = 5.430, P = 2.610 × 10^−5^), indicating the two-locus model of SLC2A1 rs4658-HSPD1 rs17730989 might be related to drug sensitivity. Then we confirmed this model in validation stage and no significant correlation was found (P = 0.340). We further explored the multi-dimensional SNP-SNP interaction. Due to the limitation of subjects’ number, the number of dimension was set from 3 to 6. We finally found that the best model was six dimension containing ARHGAP26 rs3776332, BRCA1 rs799917, ERCC6 rs2228528, NPAT rs228589, REV3L rs462779 and SLC2A1 rs4658 with testing accuracy of 0.547 CV consistency of 4/10, significant test P = 0.011 (Table 2). However, this six-locus model could not be replicated in the validation cohort (Table 2). Thus, we didn’t find a gene-gene interaction associated with drug response.

**Table 2:** Multi-dimensional gene-gene interaction in discovery stage and validation stage.

Study Component	Phenotype	Model	Training Bal. Acc.	Testing Bal. Acc.	Sign Test (*p*)	CV Consistency
Discovery	Overall toxicity					
		rs2231142-rs228589-rs2929970	0.666	0.473	4 (0.828)	2/10
		rs2228528-rs462779-rs2929970-rs189037	0.740	0.487	4 (0.828)	3/10
		rs3776332-rs2228528-rs462779-rs2977549-rs189037	0.816	0.493	5 (0.623)	5/10
		rs3776332-rs799917-rs2228528-rs462779-rs2977549-rs189037	0.885	0.565	6 (0.377)	4/10
	Hematologic toxicity					
		rs799917-rs28688207-rs2977530	0.688	0.538	6 (0.377)	6/10
		rs2231142-rs3859104-rs228589-rs2977549	0.761	0.517	4 (0.828)	2/10
		rs799917-rs2228528-rs462779-rs4658-rs189037	0.841	0.527	7 (0.172)	4/10
		rs3776332-rs799917-rs2228528-rs228589-rs462779-rs4658	0.906	0.533	5 (0.623)	5/10
	Gastrointestinal toxicity					
		rs462779-rs2929970-rs189037	0.716	0.542	6 (0.377)	5/10
		rs3776332-rs2070804-rs462779-rs189037	0.796	0.554	9 (0.012)	5/10
		rs3776332-rs799917-rs2228528-rs2977549-rs189037	0.877	0.612	10 (0.001)	9/10
		rs3776332-rs799917-rs2228528-rs4658-rs2977549-rs189037	0.922	0.593	7 (0.172)	5/10
	Sensitivity					
		rs2228528-rs28688207-rs2977530	0.719	0.547	8 (0.055)	4/10
		rs3776332-rs2228528-rs4658-rs2977549	0.801	0.501	5 (0.623)	3/10
		rs3776332-rs2228528-rs228589-rs462779-rs2977549	0.877	0.516	5 (0.623)	2/10
		rs3776332-rs799917-rs2228528-rs228589-rs462779-rs4658	0.933	0.547	9 (0.011)	4/10
Validation	Gastrointestinal toxicity					
		rs3776332-rs2070804-rs462779-rs189037	0.749	0.434	2 (0.989)	10/10
		rs3776332-rs799917-rs2228528-rs2977549-rs189037	0.706	0.504	6 (0.377)	10/10
	Sensitivity					
		rs2228528-rs28688207-rs2977530	0.530	0.471	4 (0.828)	10/10
		rs3776332-rs799917-rs2228528-rs228589-rs462779-rs4658	0.810	0.448	2 (0.989)	10/10

We next considered whether environment factors such as age, gender, smoking status, tumor histology and PS score could play a role in mediating the effects of gene-gene interaction on platinum-based chemotherapy response. Then environment factors were set as markers to investigate the association. As shown in Table 3, the two best models that identified associated with platinum-based chemotherapy sensitivity were a four dimensional model of ARHGAP26 rs3776332-ERCC6 rs2228528-SLC2A1 rs4658-histology, with testing accuracy of 0.636, CV consistency of 7/10 and significant test P = 0.001, and a five dimensional model of ARHGAP26 rs3776332-ERCC6 rs2228528-NPAT rs228589-WISP1 rs2977549-histology, with testing accuracy of 0.674, CV consistency of 7/10 and significant test P = 0.001. In the validation stage, as shown in Table 4, only the four dimensional model was verified (testing accuracy of 0.591, CV consistency of 10/10 and significant test P = 0.011). In addition, a consistent result was obtained from a second validation in the combination cohort with testing accuracy of 0.616, CV consistency of 10/10and significant test P = 0.001 (Table 4). Thus, we concluded that the four dimensional model of ARHGAP26 rs3776332-ERCC6 rs2228528-SLC2A1 rs4658-histology was significantly associated with platinum-based chemotherapeutic response.

**Table 3:** Multi-dimensional gene-environment interaction in discovery stage.

Phenotype	Model	Training Bal. Acc.	Testing Bal. Acc.	Sign Test (*p*)	CV Consistency
Overall toxicity					
	rs228589-rs2929970	0.616	0.529	7 (0.172)	3/10
	rs2228528-rs2929970-Histology	0.672	0.542	9 (0.011)	3/10
	rs2228528-rs462779-rs2929970-Histology	0.745	0.546	8 (0.055)	6/10
	rs2228528-rs462779-rs2929970-rs189037-Histology	0.824	0.559	8 (0.055)	7/10
	rs3776332-rs799917-rs2228528-rs2977549-rs189037-Histology	0.894	0.561	9 (0.011)	8/10
Hematologic toxicity					
	rs2231142-rs2070804	0.626	0.499	4 (0.828)	4/10
	rs799917-rs28688207-rs2977530	0.689	0.520	6 (0.377)	5/10
	rs2228528-rs462779-rs4658-Histology	0.762	0.475	1 (0.999)	2/10
	rs799917-rs2228528-rs462779-rs4658-rs189037	0.844	0.521	6 (0.377)	2/10
	rs3776332-rs2228528-rs228589-rs462779-rs2977549-Histology	0.914	0.473	3 (0.945)	3/10
Gastrointestinal toxicity					
	rs2977549-PS	0.649	0.532	6 (0.377)	4/10
	rs462779-rs2929970-rs189037	0.717	0.513	5 (0.623)	4/10
	rs3776332-rs2070804-rs462779-rs189037	0.798	0.525	8 (0.055)	3/10
	rs3776332-rs799917-rs2228528-rs2977549-rs189037	0.877	0.612	10 (0.001)	9/10
	rs3776332-rs799917-rs2228528-rs2977549-rs189037-Histology	0.927	0.496	4 (0.828)	3/10
Sensitivity					
	rs2228528-Histology	0.661	0.550	8 (0.055)	6/10
	rs3776332-rs4658-Histology	0.720	0.539	8 (0.055)	3/10
	rs3776332-rs2228528-rs4658-Histology	0.808	0.636	10 (0.001)	7/10
	rs3776332-rs2228528-rs228589-rs2977549-Histology	0.889	0.674	10 (0.001)	7/10
	rs3776332-rs2228528-rs228589-rs462779-rs2977549-Histology	0.941	0.605	8 (0.055)	4/10

**Table 4:** Multi-dimensional gene-environment interaction in validation stage.

Phenotype	Model	Training Bal. Acc.	Testing Bal. Acc.	Sign Test (*p*)	CV Consistency
Overall toxicity					
	rs2228528-rs2929970-Histology	0.564	0.536	8 (0.055)	10/10
	rs2228528-rs462779-rs2929970-Histology	0.582	0.473	4 (0.828)	10/10
	rs2228528-rs462779-rs2929970-rs189037-Histology	0.649	0.495	6 (0.377)	10/10
	rs3776332-rs799917-rs2228528-rs2977549-rs189037-Histology	0.717	0.495	5 (0.623)	10/10
Gastrointestinal toxicity					
	rs3776332-rs2070804-rs462779-rs189037	0.749	0.434	2 (0.989)	10/10
	rs3776332-rs799917-rs2228528-rs2977549-rs189037	0.706	0.504	6 (0.377)	10/10
Sensitivity					
	rs4658-rs3776332-rs2228528-Histology	0.665	0.591	9 (0.011)	10/10
	rs3776332-rs2228528-rs228589-rs2977549-Histology	0.685	0.562	8 (0.055)	10/10
	rs3776332-rs2228528-rs228589-rs2977549-rs462779-Histology	0.760	0.500	4 (0.828)	10/10

### Influence of gene-gene and gene-environment interaction to platinum-based chemotherapeutic ADR

Next, we studied the association of interactions with platinum-based chemotherapeutic toxicity. Primarily, we also explored the paired gene-gene interaction. Three SNP-SNP pairs that found to be significantly associated with overall toxicity in the discovery stage included ABCG2 rs2231142-CES5A rs3859104 (adjusted OR = 8.040, P = 4.350 × 10^−5^), HSPD1 rs17730989-SUMF1 rs2633851 (adjusted OR = 0.084, P = 4.670 × 10^−5^) and ARHGAP26 rs3776332-HSPB1 rs2070804 (adjusted OR = 6.380, P = 5.310 × 10^−5^). Then we further try to verify them in 788 patients in the validation cohort. It was interesting to note that ABCG2 rs2231142-CES5A rs3859104 model was successfully verified (adjusted OR = 1.870, P = 0.011). As for the multi-dimensional SNP-SNP interactions, we discovered that even the best model had no association with the overall toxicity (Table 2). Afterwards, we explored gene-environment interaction, a six dimensional model including ARHGAP26 rs3776332, BRCA1 rs799917, ERCC6 rs2228528, WISP1 rs2977549, ATM rs189037 and histology was identified to be significantly associated with overall toxicity (testing of accuracy 0.561, CV of consistency 8/10, significant test P = 0.011) (Table 3). However, this model was not validated (Table 4). Thus, the paired interaction of ABCG2 rs2231142-CES5A rs3859104 was associated with chemotherapy overall toxicity.

Considering the overall toxicity contained hematologic and gastrointestinal toxicity, we thus investigated the interactions in these two subgroup phenotypes. Interaction between HSPD1 rs17730989-SUMF1 rs2633851 and HSPD1 rs28688207-BRCA1 rs799917 pairs was found to be significantly related to hematologic toxicity. The HSPD1 rs17730989-SUMF1 rs2633851 pair was successfully verified in validation cohorts (Fig. 1 and Supplementary Table S3). But the multi-dimensional gene-gene and gene-environment models had no relationship to hematologic toxicity (Tables 2 and 3).

For gastrointestinal toxicity, interactions of the REV3L rs462779-ATM rs189037 pair (adjusted OR = 0.272, P = 3.900 × 10^−5^), ATM rs189037-ERCC6 rs2228528 pair (adjusted OR = 6.780, P = 7.220 × 10^−5^) and REV3L rs462779-NPAT rs228589 pair (adjusted OR = 0.306, P = 9.370 × 10^−5^) were found to be significantly associated with this phenotype (Fig. 1 and Supplementary Table S3). A five dimensional model of ARHGAP26 rs3776332-BRCA1 rs799917-ERCC6 rs2228528-WISP1 rs2977549 -ATM rs189037 was also associated with gastrointestinal toxicity (Table 2). However, none of them could be verified in the validation stage (Table 2 and Supplementary Table S3). In addition, no significant gene-environment interaction was identified associated with gastrointestinal toxicity (Table 3).

In summary, the paired interaction of ABCG2 rs2231142-CES5A rs3859104 was associated with overall toxicity. And the HSPD1 rs17730989-SUMF1 rs2633851 pair was significantly related to hematologic toxicity.

## Discussion

In this study, we investigated the association of gene-gene interaction with chemotherapy response and toxicity. The four-dimensional model of ARHGAP26 rs3776332-ERCC6 rs2228528-SLC2A1 rs4658-histology, paired interactions of ABCG2 rs2231142-CES5A rs3859104 and HSPD1 rs17730989- SUMF1 rs2633851 were found to be significantly associated with platinum-based chemotherapeutic response, overall and hematological toxicities respectively. All these interactions found in the discovery stage were successfully verified in validation stage.

In the current study, we found that some SNPs previously identified “negative” were in fact significantly related to phenotype in the form of SNP-SNP pairs. Many SNPs analyzed in this study were reported to have no association with platinum-based chemotherapy sensitivity and toxicity^13, 16–18^. These studies only focused on a single SNP which overlooked possible interactions between inter-SNPs. Gene-gene interaction takes genetic context into account, and the “missing heritability” partly attribute to the low power to detect gene-gene interactions mentioned. So gene-gene interaction acts as an indispensable approach of studying how SNPs influence phenotype. In addition, environment factors were not taken into account in the univariate studies. The factors alone didn’t have so notable association with platinum-based chemotherapy sensitivity. When considering gene-environment interaction, we can found that environment also played important role in drug response. In conclusion, the analysis of gene-gene and gene-environment interaction could further discover SNPs that were associated with platinum-based chemotherapeutic response and toxicity. The strategy of analyzing gene-gene and gene-environment interaction may hold potential value to predict chemotherapeutic response and toxicity in NSCLC patients.

The effects of SNP-SNP interactions on chemotherapeutic response and toxicity could be partly explained by the specific function of these genes. The rs2231142 is a non-synonymous variant in ABCG2 (C421A, encoding Q141K, Gln141Lys), which is a member of ATP -binding cassette (ABC) transporters super family^19, 20^. And it is reported that the ABCG2C421A variant is associated with severe thrombocytopenia^21^. CES5A is one of the five subfamilies of CEs and generally expressed in the liver. CES enzymes mediate drug hydrolysis and the metabolic products are, to some extent, responsible for hepatotoxicity and nephrotoxicity or hematic toxicity^22^. In a word, ABCG2 and CES5A are all associated with platinum-based chemotherapy toxicity, so when we take them both into consideration, the predication of toxicity may be improved. In terms of chemotherapy-induced hematologic toxicity, Heat Shock Proteins (HSPs) are the major chaperones mediating (re) folding of proteins, and Sulfatase modifying factor 1 gene (Sumf1) can activate the catalytic site of SGSH^23^.

When exploring potential association with platinum-based chemotherapy response, Rho GTPase activating protein 26 (ARHGAP26) is a negative regulator of the Rho family that converts the small G proteins RhoA and Cdc42 to their inactive GDP-bound forms, and is associated with gastric cancer^24, 25^. Functional Rho can regulate activity of the c-fos promoter, which can enhance cell survival while leads to apoptosis in high concentrations^26, 27^. Solute carrier family 2 facilitated glucose transporter member 1 (SLC2A1) supply cells with glucose by facilitating diffusion of glucose molecules across the plasma membrane when the cellular glucose concentration is low^28^. DNA repair gene ERCC6 is an important caretaker of the overall genome stability, and different genotypes of rs2228528 were associated with susceptibility^29^. In conclusion, these SNPs identified in the study are related to platinum-based chemotherapeutic response and toxicity, which is possibly a result of their impacts on function of relevant genes. A SNP in a gene can cause the change of phenotype in the process of platinum-based chemotherapy. The accumulated effect of two or more SNPs may perform a more important role in this process as our results indicated. Traditionally, histology was regarded as an important factor in treatment decisions in NSCLC patients. However, the histology couldn’t predict platinum-based chemotherapeutic response and toxicity precisely owing to its heterogeneity. When we took genes into consideration, the predictive accuracy was improved. In other words, environmental factors combined with genes can achieve better predictive outcomes.

It’s undeniable that the there are some limitation in our study. First, We did Bonferroni correction in our multiple tests and most of the results were statistically significant except rs17730989 HSPD1 - rs2633851 SUMF1 (OR is 0.233 and p-value is 0.018). However, this result was verified in independent validation cohort (OR is 0.570 and p-value is 0.018), so we regarded it as statistical significance and kept it in this article. Moreover, the GI toxicity between discovery cohort (17.35%) and validation cohort (8.38%) was inconsistent. We think that it may be one of the reasons why we failed to validate GI models.

In a word, this study aimed to propose a feasible approach to explore the association with SNPs and platinum-based chemotherapy induced susceptibility and toxicity. The combined effect of gene and environment via gene-gene and gene-environment interaction may help to predict the phenotype of NSCLC patients. And gene-gene interaction as well as gene-environment model may be indispensable in phenotype prediction in the future.

## Subjects and Methods

### Subjects

The current two-stage clinical investigation enrolled 490 and 788 NSCLC patients for the discovery and validation cohort, respectively. The clinical characteristics of all subjects were summarized in Table 1. All patients provided written informed consent in compliance with the code of ethics of the World Medical Association (Declaration of Helsinki) before this study was launched. The study protocol was approved by the Ethics Committee of Xiangya School of Medicine, Central South University (Registration Number: CTXY-110008-1). The study was also applied for clinical admission in the Chinese Clinical Trial Registry with Registration Number of ChiCTR-ROC-14005699.

Patients eligible for the study had to meet the following criteria: (1) diagnosed as NSCLC histologically and confirmed as a primary tumor; (2) receiving neither surgery nor radiation therapy before or during chemotherapy; (3) treated with platinum-based chemotherapy regimens containing platinum + gemcitabine (GP), platinum + docetaxel (DP), platinum + etoposide (EP), platinum + paclitaxel (TP), platinum + pemetrexed (PP), and other platinum-based chemotherapy (platinum + irinotecan, platinum + vinorelbine) at least two cycles; (4) assessed treatment efficacy before the third chemotherapy cycle. Patients who have pregnancy, lactation, active infection, symptomatic brain or leptomeningeal metastases, and other previous or concomitant malignancies were excluded.

### Data collection

We collected clinical data of all patients, including age, gender, smoking status, tumor histology and clinical stage. After two cycles of treatment, the response to chemotherapy was evaluated following the Response Evaluation Criteria in Solid Tumors (RECIST) guidelines. The curative effect was classified as complete response (CR), partial response (PR), stable disease (SD), and progressive disease (PD)^30^. We defined CR and PR as platinum-sensitive phenotypes, SD and PD as platinum-resistant phenotypes. According to the National Cancer Institute Common Toxicity Criteria 3.0 (NCI-CTC 3.0), toxicity was evaluated when participants receiving first two cycles of chemotherapy. And we classified toxicity into hematologic toxicity (anemia, leukopenia, neutropenia and thrombocytopenia) and gastrointestinal toxicity. Drug toxicity was classified into grade 0–4, grade 0–2 was considered as low-toxicity and grade 3 or 4 was considered as high-toxicity. Either gastrointestinal toxicities or hematologic toxicities that reach to grade 3 or 4 were considered as overall toxicity as CTCAE guide.

### SNPs selection, sample preparation and genotyping

Most of the SNPs were selected as previously described^13^. In brief, genes associated with platinum response or toxicity were selected, a total of 504 SNPs with a minor allele frequency (MAF) ≥ 0.05 were genotyped in the discovery stage (Supplementary Table S1). The distribution of these SNPs located genes was shown in Supplementary Fig. S1. After a screening process, we selected 16 SNPs that were associated with chemotherapy sensitivity and toxicity for further validation.

Genomic DNA of all subjects was extracted from approximate 5 ml peripheral blood using Genomic DNA Purification Kit (Promega, Madison, Wisconsin, USA) according to the standard protocol, and was stored at −20 °C until use. We obeyed the experimental operating practices and qualities of all DNA samples were verified by agarose gel electrophoresis. All genotyping were conducted by Sequenom’s Mass ARRAY system (Sequenom, San Diego, California, USA).

### Statistical analysis

Continuous variables were presented as means ± SD and analyzed by the two-sample t-test. Noncontiguous variables in different groups were compared using the χ^2^ test. To investigate the influence of gene-gene and gene-environment interactions on platinum-based chemotherapy response and toxicity, PLINK and generalized multifactor dimensionality reduction (GMDR) software were employed^31, 32^. All of the statistical analysis was planned and conducted in accordance with relevant guidelines. In this paper, false positive rate (FPR) and true positive rate (TPR) were employed to evaluate the model prediction accuracy. Cross-validation consistency was used to assess the quality of the model. And the sign test was used to evaluate the model statistically significance. Models with the maximum testing accuracy and cross-validation consistency (CVC) were regarded as the best interaction model. All P-value was two-tailed and P < 0.05 was considered as statistically significant. The flow diagram of the analysis was showed in Supplementary Fig. S2.

## Electronic supplementary material

Supplementary Information

## References

[1] Siegel RL, Miller KD, Jemal A (2016). Cancer statistics, 2016. CA: a cancer journal for clinicians.

[2] Chen W (2016). Cancer statistics in China, 2015. CA: a cancer journal for clinicians.

[3] Torre LA (2015). Global cancer statistics, 2012. CA: a cancer journal for clinicians.

[4] Ettinger DS (2015). Non–small cell lung cancer, version 6.2015. Journal of the National Comprehensive Cancer Network.

[5] Huang Q (2013). Two novel functional single nucleotide polymorphisms of ADRB3 are associated with type 2 diabetes in the Chinese population. The Journal of Clinical Endocrinology & Metabolism.

[6] Yin J-Y, Huang Q, Zhao Y-C, Zhou H-H, Liu Z-Q (2012). Meta-analysis on pharmacogenetics of platinum-based chemotherapy in non small cell lung cancer (NSCLC) patients. PloS one.

[7] Yin JY (2011). ABCC1 polymorphism Arg723Gln (2168G > A) is associated with lung cancer susceptibility in a Chinese population. Clinical and Experimental Pharmacology and Physiology.

[8] Yin J-Y (2009). Characterization and analyses of multidrug resistance-associated protein 1 (MRP1/ABCC1) polymorphisms in Chinese population. Pharmacogenetics and genomics.

[9] Relling MV, Evans WE (2015). Pharmacogenomics in the clinic. Nature.

[10] Xu, H. *et al*. Common variants in ACYP2 influence susceptibility to cisplatin-induced hearing loss. *Nature genetics*, doi:10.1038/ng.3217 (2015). 10.1038/ng.3217 PMC4358157 25665007

[11] Ross CJ (2009). Genetic variants in TPMT and COMT are associated with hearing loss in children receiving cisplatin chemotherapy. Nature genetics.

[12] Manolio TA (2009). Finding the missing heritability of complex diseases. Nature.

[13] Yin J-Y (2016). Prediction models for platinum-based chemotherapy response and toxicity in advanced NSCLC patients. Cancer letters.

[14] Cordell HJ (2009). Detecting gene-gene interactions that underlie human diseases. *Nature reviews*. Genetics.

[15] Prabhu S, Pe’er I (2012). Ultrafast genome-wide scan for SNP-SNP interactions in common complex disease. Genome research.

[16] Chen J (2014). WISP1 polymorphisms contribute to platinum-based chemotherapy toxicity in lung cancer patients. International journal of molecular sciences.

[17] Wang Y (2014). The association of transporter genes polymorphisms and lung cancer chemotherapy response. PloS one.

[18] Yin JY (2015). Association of positively selected eIF3a polymorphisms with toxicity of platinum-based chemotherapy in NSCLC patients. Acta pharmacologica Sinica.

[19] Tian C (2012). Common variants in ABCB1, ABCC2 and ABCG2 genes and clinical outcomes among women with advanced stage ovarian cancer treated with platinum and taxane-based chemotherapy: a Gynecologic Oncology Group study. Gynecologic oncology.

[20] Gozzi GJ (2015). Phenolic indeno [1, 2-b] indoles as ABCG2-selective potent and non-toxic inhibitors stimulating basal ATPase activity. Drug design, development and therapy.

[21] Low S-K (2016). Association Study of a Functional Variant on ABCG2 Gene with Sunitinib-Induced Severe Adverse Drug Reaction. PloS one.

[22] Shimizu M, Fukami T, Nakajima M, Yokoi T (2014). Screening of specific inhibitors for human carboxylesterases or arylacetamide deacetylase. Drug Metabolism and Disposition.

[23] Tardieu M (2014). Intracerebral administration of adeno-associated viral vector serotype rh. 10 carrying human SGSH and SUMF1 cDNAs in children with mucopolysaccharidosis type IIIA disease: results of a phase I/II trial. Human gene therapy.

[24] Yao F (2015). Recurrent Fusion Genes in Gastric Cancer: CLDN18-ARHGAP26 Induces Loss of Epithelial Integrity. Cell reports.

[25] Wang Q (2013). ADAR1 regulates ARHGAP26 gene expression through RNA editing by disrupting miR-30b-3p and miR-573 binding. RNA.

[26] Yeung CK, Chan KP, Chiang SW, Pang CP, Lam DS (2003). The toxic and stress responses of cultured human retinal pigment epithelium (ARPE19) and human glial cells (SVG) in the presence of triamcinolone. Investigative ophthalmology & visual science.

[27] Hill CS, Wynne J, Treisman R (1995). The Rho family GTPases RhoA, Racl, and CDC42Hsregulate transcriptional activation by SRF. Cell.

[28] Ooi AT, Gomperts BN (2015). Molecular pathways: targeting cellular energy metabolism in cancer via inhibition of SLC2A1 and LDHA. Clinical Cancer Research.

[29] Chiu C-F (2008). A novel single nucleotide polymorphism in ERCC6 gene is associated with oral cancer susceptibility in Taiwanese patients. Oral oncology.

[30] Eisenhauer EA (2009). New response evaluation criteria in solid tumours: revised RECIST guideline (version 1.1). European journal of cancer.

[31] Lou XY (2007). A generalized combinatorial approach for detecting gene-by-gene and gene-by-environment interactions with application to nicotine dependence. American journal of human genetics.

[32] Purcell S (2007). PLINK: a tool set for whole-genome association and population-based linkage analyses. The American Journal of Human Genetics.

