# Supplementary material for: Gene-gene and gene-environment interactions influence platinum-based chemotherapy response and toxicity in non-small cell lung cancer patients
Source: Sci Rep. 2017 Jul 11;7:5082. doi: 10.1038/s41598-017-05246-8 (PMC5505954; doi:10.1038/s41598-017-05246-8)
Supplement: Supplementary file 1 — Supplementary Information [file 41598_2017_5246_MOESM1_ESM.doc]

**Gene-gene and gene-environment interactions influence** **platinum-based chemotherapy response and toxicity in non-small cell lung cancer patients**

Jia-Jia Cui^1#^, Lei-Yun Wang^1#^, Tao Zhu^1^, Wei-Jing Gong^1^, Hong-Hao Zhou^1^, Zhao-Qian Liu^1^, Ji-Ye Yin^1*^

^1^Department of Clinical Pharmacology, Xiangya Hospital, Central South University, Changsha 410008; P. R. China; Institute of Clinical Pharmacology, Central South University; Hunan Key Laboratory of Pharmacogenetics, Changsha 410078; P. R. China

*To whom correspondence should be addressed: Professor Ji-Ye Yin, Department of Clinical Pharmacology, Xiangya Hospital, Central South University, Changsha 410008; P. R. China; Institute of Clinical Pharmacology, Hunan Key Laboratory of Pharmacogenetics, Central South University, Changsha, Hunan 410078, P. R. China. Tel: +86 731 84805380, Fax: +86 731 82354476, E-mail: [yinjiye@csu.edu.cn](mailto:yinjiye@csu.edu.cn)

^#^ The two authors contributed equally to this work.

**
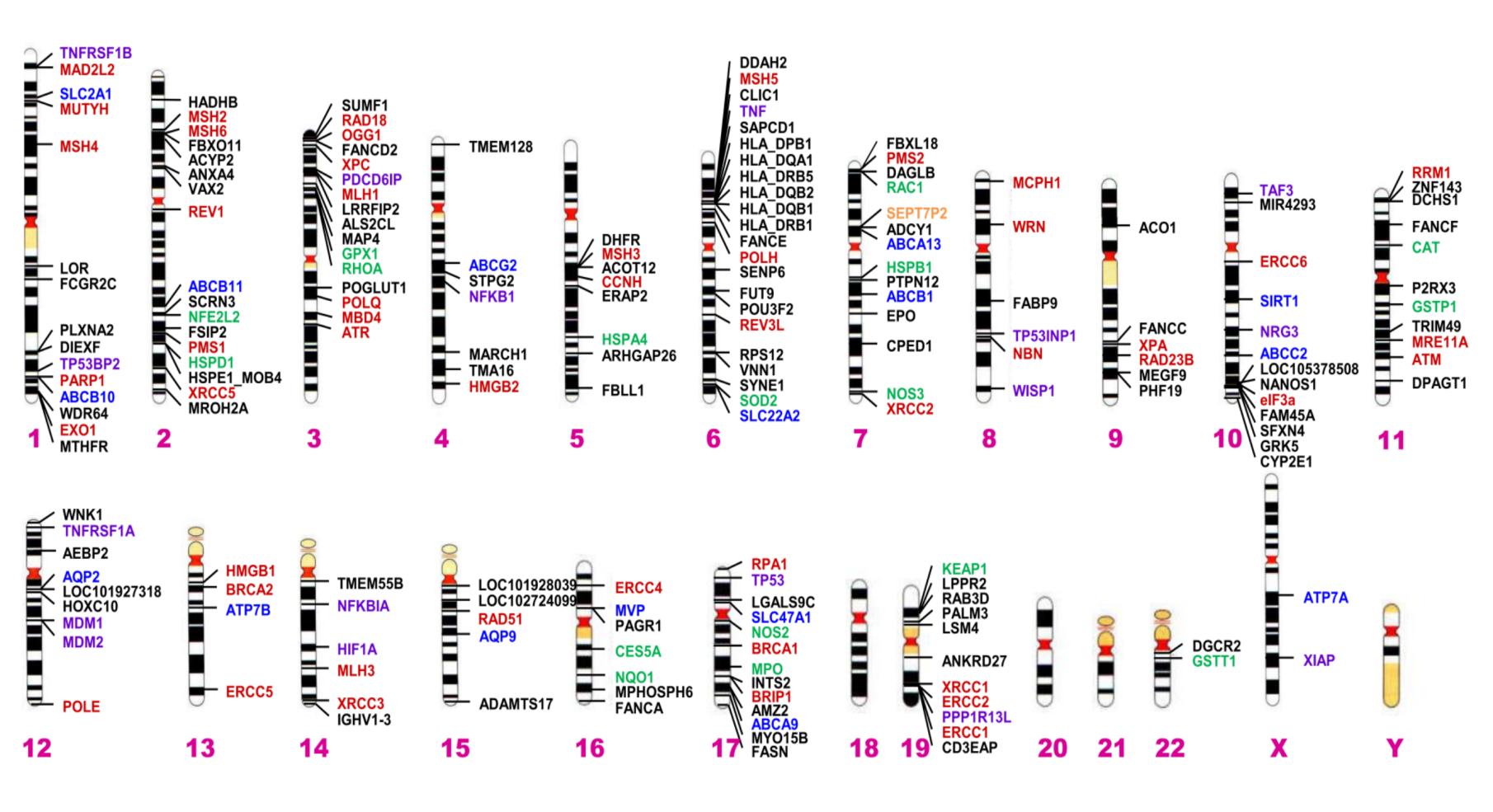
**

**Figure S1 Distribution of selected genes on chromosome.** All the genes selected in this study are labeled in corresponding chromosomes. Red represents DNA repair, green represents detoxification, purple represents apoptosis, blue represents transporters and black represents other pathways.


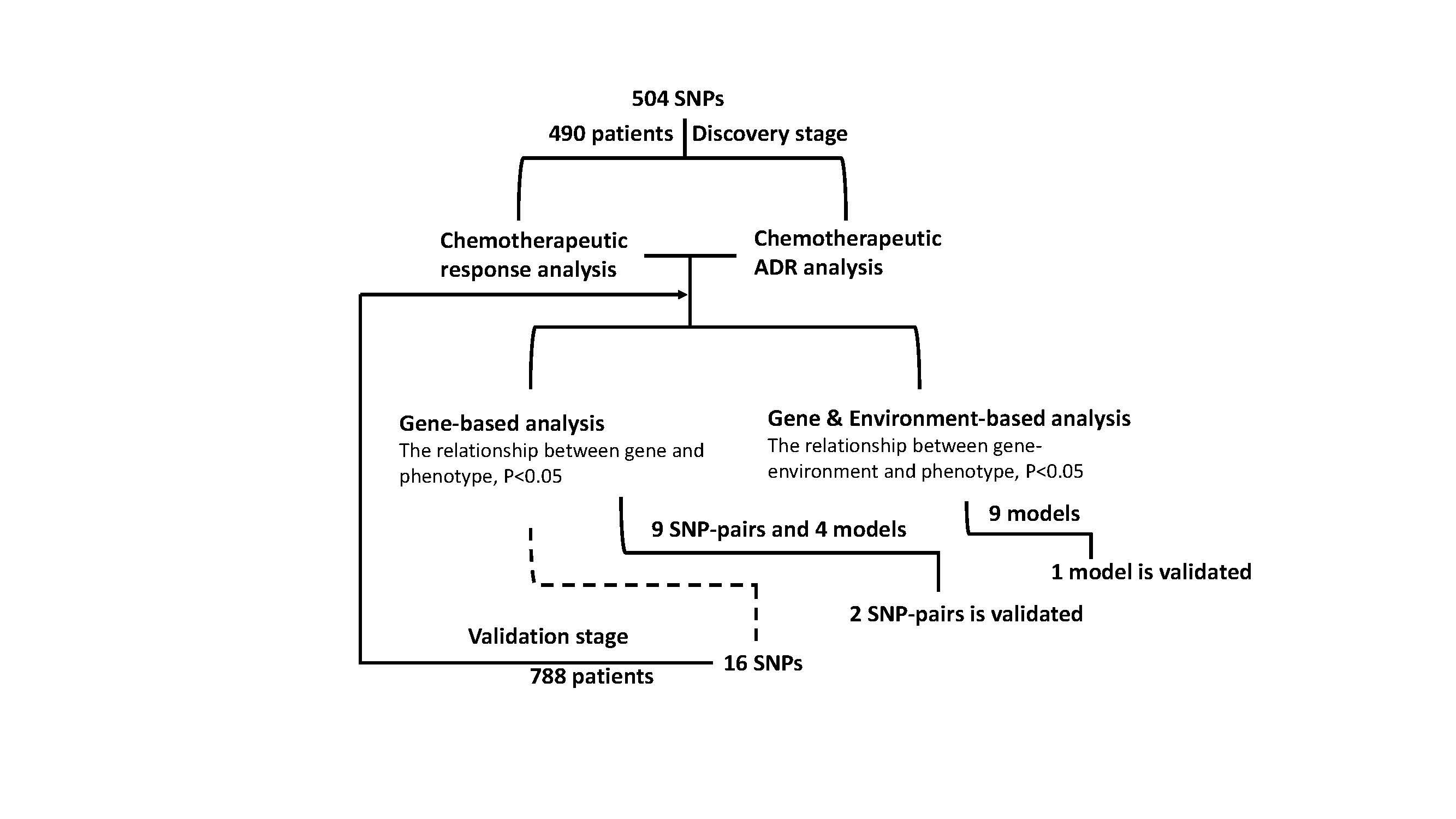


**Figure S2. Flow diagram of the data analysis in the study.** Process flow diagram of data analysis in this study. We firstly explore the relationship between genes and platinum-based chemotherapy response and toxicity. And then we explored the interactions between genes and environmental factors based on it. The 16 SNPs were proved to be remarkably associated with chemotherapy response and toxicities in step 1 and indicated by a dotted line. 9 paired gene-gene interaction and 4 multi-dimensional gene-gene models were screened for validated and 2 paired gene-gene interaction were validated in the validation cohort as Fig.1 presented. Then we studied association of gene-environment interactions with platinum-based chemotherapeutic response and toxicity, we found 9 gene-environment models in the discovery stage and 1 model was statistically significant.

**Table S1: Characteristics and annotation of all genotyped SNPs**

| **SNPs** | **Chr** | **Genes** | **Localization** | **Chromosome position** | **Transcript** | **cDNA position** | **Amino acid position** | **Amino acid change** |
| --- | --- | --- | --- | --- | --- | --- | --- | --- |
| rs735943 | 1 | EXO1 | Exon | 242030151 | NM_003686 | 1375 | 354 | H>R |
| rs2233004 | 1 | MAD2L2 | 5' near gene | 11742074 | NM_006341 | - | - | - |
| rs746218 | 1 | MAD2L2 | 5' near gene | 11743124 | NM_006341 | - | - | - |
| rs1047840 | 1 | EXO1 | Exon | 242042301 | NM_003686 | 279 | 589 | E>K |
| rs1776148 | 1 | EXO1 | Exon | 242042545 | NM_003686 | 2323 | 67 | E>G |
| rs2185383 | 1 | PLXNA2 | 5' near gene | 208779683 | NM_025179 | - | - | - |
| rs2185383 | 1 | PLXNA2 | 5' near gene | 208779683 | NM_025179 | - | - | - |
| rs2233006 | 1 | MAD2L2 | 5' near gene | 11741796 | NM_006341 | - | - | - |
| rs3219489 | 1 | MUTYH | Exon | 45797505 | NM_001048171 | 1188 | 324 | Q>H |
| rs1136410 | 1 | PARP1 | Exon | 226555302 | NM_001618 | 2456 | 762 | V>A |
| rs1061622 | 1 | TNFRSF1B | Exon | 12252955 | NM_001066 | 676 | 196 | M>R |
| rs1053513 | 1 | ABCB10 | 3'-UTR | 229652587 | NM_012089 | 3599 | - | - |
| rs17739 | 1 | TP53BP2 | 3'-UTR | 223967953 | NM_001031685 | 4299 | - | - |
| rs7539638 | 1 | DIEXF | 3'-UTR | 210025751 | NM_014388 | 3327 | - | - |
| rs1146642 | 1 | MSH4 | 5' near gene | 76261434 | NM_002440 | - | - | - |
| rs3806162 | 1 | MSH4 | 5' near gene | 76261995 | NM_002440 | - | - | - |
| rs5745532 | 1 | MSH4 | Intron | 76362465 | NM_002440 | - | - | - |
| rs3738514 | 1 | SLC2A1 | 5' near gene | 43426591 | NM_006516 | - | - | - |
| rs1385129 | 1 | SLC2A1 | Exon | 43408966 | NM_006516 | 57 |  | A>A |
| rs3806400 | 1 | SLC2A1 | 5' near gene | 43426378 | NM_006516 | - | - | - |
| rs4658 | 1 | SLC2A1 | 3'-UTR | 43392250 | NM_006516 | 2466 | - | - |
| rs841844 | 1 | SLC2A1 | 3' near gene | 43387302 | NM_006516 | - | - | - |
| rs3820589 | 1 | SLC2A1 | 5' near gene | 43426044 | NM_006516 | - | - | - |
| rs11205255 | 1 | LOR | 3' near gene | 153234602 | NM_000427 | - | - | - |
| rs61801822 | 1 | FCGR2C | Intron | 161565324 | NM_201563 | - | - | - |
| rs67170285 | 1 | WDR64 | Intron | 241838057 | NM_144625 | - | - | - |
| rs1801133 | 1 | MTHFR | Exon | 11856378 | NM_005957 | 894 | 222 | A>V |
| rs1042821 | 2 | MSH6 | Exon | 48010488 | NM_000179 | 268 | 39 | G>E |
| rs2303425 | 2 | MSH2 | 5'-UTR | 47630213 | NM_000251 | 8 | - | - |
| rs6706649 | 2 | NFE2L2 | 5' near gene | 178130071 | NM_001145412 | - | - | - |
| rs3087386 | 2 | REV1 | Exon | 100055506 | NM_001037872 | 982 | 257 | F>S |
| rs2303428 | 2 | MSH2 | Intron | 47703500 | NM_000251 | - | - | - |
| rs35652124 | 2 | NFE2L2 | 5' near gene | 178130073 | NM_001145412 | - | - | - |
| rs5742933 | 2 | PMS1 | 5'-UTR | 190649316 | NM_000534 | 56 | - | - |
| rs6721961 | 2 | NFE2L2 | 5' near gene | 178130037 | NM_001145412 | - | - | - |
| rs6941 | 2 | XRCC5 | 3-UTR | 216205929 | - | - | - | - |
| rs1051677 | 2 | XRCC5 | 3'-UTR | 217070248 | NM_021141 | 2662 | - | - |
| rs2440 | 2 | XRCC5 | 3'-UTR | 217070766 | NM_021141 | 318 | - | - |
| rs495714 | 2 | ABCB11 | 3'-UTR | 169779764 | NM_003742 | 446 | - | - |
| rs10191478 | 2 | MSH2 | Intron | 47645249 | NM_000251 | - | - | - |
| **Table S1.** (Continued) | | | | | | | | |
| rs2020910 | 2 | MSH6 | Exon | 48030692 | NM_000179 | 3458 | 112 | T>T |
| rs2348244 | 2 | MSH6 | Intron | 48019485 | NM_000179 | - | - | - |
| rs2710163 | 2 | MSH6 | 3' near gene | 48035673 | NM_000179 | - | - | - |
| rs6713506 | 2 | FBXO11 | Intron | 48038331 | NM_001190274 | - | - | - |
| rs6742522 | 2 | FBXO11 | Intron | 48038944 | NM_001190274 | - | - | - |
| rs17730989 | 2 | HSPD1 | Intron | 198362524 | NM_002156 | - | - | - |
| rs1981929 | 2 | MSH2 | Intron | 47672569 | NM_000251 | - | - | - |
| rs3732190 | 2 | FBXO11 | Intron | 48037593 | NM_001190274 | - | - | - |
| rs4608577 | 2 | MSH2 | Intron | 47703984 | NM_000251 | - | - | - |
| rs6544991 | 2 | MSH2 | Intron | 47712780 | NM_000251 | - | - | - |
| rs7602094 | 2 | MSH2 | Intron | 47673515 | NM_000251 | - | - | - |
| rs1051685 | 2 | XRCC5 | 3'-UTR | 217070376 | NM_021141 | 279 | - | - |
| rs13019654 | 2 | MSH2 | Intron | 47689217 | NM_000251 | - | - | - |
| rs13386066 | 2 | HSPE1_MOB4 | Intron | 198371687 | NM_001202485 | - | - | - |
| rs2605039 | 2 | HSPE1_MOB4 | 5' near gene | 198362851 | NM_001202485 | - | - | - |
| rs12999145 | 2 | MSH2 | Intron | 47686686 | NM_000251 | - | - | - |
| rs3136329 | 2 | MSH6 | Intron | 48024876 | NM_000179 | - | - | - |
| rs4952887 | 2 | MSH2 | Intron | 47646968 | NM_000251 | - | - | - |
| rs2290158 | 2 | SCRN3 | Intron | 175287778 | NM_001193528 | - | - | - |
| rs10496029 | 2 | ACYP2 | Intron | 54399058 | NM_138448 | - | - | - |
| rs10175949 | 2 | MROH2A | Intron | 234712921 | NM_001287395 | - | - | - |
| rs60558096 | 2 | ACYP2 | Intron | 54398837 | NM_138448 | - | - | - |
| rs2234500 | 2 | VAX2 | Exon | 71160222 | NM_012476 | 793 | 254 | P>R |
| rs36004074 | 2 | FSIP2 | Exon | 186665432 | NM_173651 | 11666 | 3889 | G>E |
| rs3732268 | 2 | ANXA4 | Intron | 70046262 | NM_001153 | - | - | - |
| rs62128457 | 2 | HADHB | 5' near gene | 26467347 | NM_000183 | - | - | - |
| rs1052133 | 3 | OGG1 | Exon | 9798773 | NM_002542 | 132 | 326 | S>C |
| rs1800734 | 3 | MLH1 | 5'-UTR | 37034946 | NM_000249 | 16 | - | - |
| rs2228000 | 3 | XPC | Exon | 14199887 | NM_001145769 | 1489 | 462 | A>V |
| rs2229032 | 3 | ATR | Exon | 142178144 | NM_001184 | 7396 | 2425 | R>Q |
| rs10342 | 3 | MBD4 | Exon | 129155670 | NM_001276270 | 1163 | 273 | A>S|T |
| rs140693 | 3 | MBD4 | Exon | 129155451 | NM_001276270 | 1382 | 346 | E>K |
| rs2227928 | 3 | ATR | Exon | 142281612 | NM_001184 | 754 | 211 | M>T |
| rs2228001 | 3 | XPC | Exon | 14187449 | NM_001145769 | 288 | 92 | Q>K |
| rs2272125 | 3 | FANCD2 | Exon | 10138069 | NM_001018115 | 4176 | 1366 | L>L |
| rs373572 | 3 | RAD18 | Exon | 8955389 | NM_020165 | 995 | 32 | R>Q |
| rs1050450 | 3 | GPX1 | Exon | 49394834 | NM_000581 | 679 | 2 | P>L |
| rs3218649 | 3 | POLQ | Exon | 121208833 | NM_199420 | 374 | 982 | T>R |
| rs3811699 | 3 | GPX1 | 5' near gene | 49396360 | NM_000581 | - | - | - |
| rs1540354 | 3 | MLH1 | Intron | 37044489 | NM_000249 | - | - | - |
| rs9852378 | 3 | MLH1 | 5'-UTR | 37038771 | NM_001258274 | 33 | - | - |
| rs10849 | 3 | LRRFIP2 | 3'-UTR | 37095070 | NM_001134369 | 197 | - | - |
| rs9878943 | 3 | RHOA | Intron | 49434654 | NM_001664 | - | - | - |
| **Table S1.** (Continued) | | | | | | | | |
| rs749072 | 3 | LRRFIP2 | Intron | 37096024 | NM_001134369 | - | - | - |
| rs12487651 | 3 | ALS2CL | Intron | 46714708 | NM_001190707 | - | - | - |
| rs12488699 | 3 | PDCD6IP | Intron | 33866868 | NM_001162429 | - | - | - |
| rs2276713 | 3 | POGLUT1 | Intron | 119205541 | NM_152305 | - | - | - |
| rs1060407 | 3 | MAP4 | Exon | 47958037 | NM_001134364 | 186 | 427 | S>Y |
| rs2633851 | 3 | SUMF1 | 3'-UTR | 4403817 | NM_001164674 | 198 | - | - |
| rs3792594 | 3 | PDCD6IP | Exon | 33877626 | NM_001162429 | 198 | 314 | A>T |
| rs615961 | 4 | STPG2 | Intron | 98737204 | NM_174952 | - | - | - |
| rs2231142 | 4 | ABCG2 | Exon | 89052323 | NM_001257386 | 769 | 141 | Q>K |
| rs230529 | 4 | NFKB1 | Intron | 103457418 | NM_001165412 | - | - | - |
| rs1585215 | 4 | NFKB1 | Intron | 103444474 | NM_001165412 | - | - | - |
| rs4648068 | 4 | NFKB1 | Intron | 103518305 | NM_001165412 | - | - | - |
| rs1448784 | 4 | ABCG2 | 3'-UTR | 89012320 | NM_001257386 | 3372 | - | - |
| rs6832850 | 4 | HMGB2 | 3' near gene | 174249593 | NM_002129 | - | - | - |
| rs7686909 | 4 | HMGB2 | 5' near gene | 174257693 | NM_002129 | - | - | - |
| rs6816526 | 4 | 42430 | 3'-UTR | 164449273 | NM_001166373 | 1679 | - | - |
| rs13130399 | 4 | 42430 | Exon | 164466824 | NM_001166373 | 677 | 165 | A>A |
| rs1561736 | 4 | TMA16 | Exon | 164440581 | NM_018352 | 88 | 176 | I>T |
| rs2916467 | 4 | TMEM128 | 5'-UTR | 4249415 | NM_032927 | 52 | - | - |
| rs26279 | 5 | MSH3 | Exon | 80168937 | NM_002439 | 3213 | 145 | A>T |
| rs1650697 | 5 | DHFR | 5'-UTR | 79950781 | NM_000791 | 2 | - | - |
| rs2230641 | 5 | CCNH | Exon | 86695274 | NM_001199189 | 1162 | 217 | V>A|D |
| rs3776332 | 5 | ARHGAP26 | Intron | 142441514 | NM_001135608 | - | - | - |
| rs6151627 | 5 | MSH3 | Intron | 79965536 | NM_002439 | - | - | - |
| rs394592 | 5 | MSH3 | 3'-UTR | 80172317 | NM_002439 | 413 | - | - |
| rs26784 | 5 | MSH3 | Intron | 80049892 | NM_002439 | - | - | - |
| rs4616886 | 5 | HSPA4 | Intron | 132423764 | NM_002154 | - | - | - |
| rs7709909 | 5 | MSH3 | Intron | 80001170 | NM_002439 | - | - | - |
| rs1650665 | 5 | MSH3 | Intron | 79962578 | NM_002439 | - | - | - |
| rs245340 | 5 | MSH3 | Intron | 80161500 | NM_002439 | - | - | - |
| rs245346 | 5 | MSH3 | Intron | 80156059 | NM_002439 | - | - | - |
| rs26778 | 5 | MSH3 | Intron | 80035750 | NM_002439 | - | - | - |
| rs3088225 | 5 | HSPA4 | 5' near gene | 132387260 | NM_002154 | - | - | - |
| rs3816729 | 5 | MSH3 | Intron | 80040532 | NM_002439 | - | - | - |
| rs6151670 | 5 | MSH3 | Intron | 79983215 | NM_002439 | - | - | - |
| rs6151892 | 5 | MSH3 | Intron | 80118881 | NM_002439 | - | - | - |
| rs6151914 | 5 | MSH3 | Intron | 80150881 | NM_002439 | - | - | - |
| rs2549794 | 5 | ERAP2 | Intron | 96244549 | NM_001130140 | - | - | - |
| rs3733977 | 5 | FBLL1 | Intron | 167957554 | NR_024356 | - | - | - |
| rs12654410 | 5 | FBLL1 | Intron | 167957055 | NR_024356 | - | - | - |
| rs13163821 | 5 | ACOT12 | Intron | 80655709 | NM_130767 | - | - | - |
| rs4880 | 6 | SOD2 | Exon | 160113872 | NM_000636 | 21 | 16 | V>A |
| rs465646 | 6 | REV3L | 3'-UTR | 111620758 | NM_001286431 | 1547 | - | - |
| **Table S1.** (Continued) | | | | | | | | |
| rs6941583 | 6 | POLH | Exon | 43582091 | NM_006502 | 2243 | 647 | M>L |
| rs3823434 | 6 | FANCE | Exon | 35426175 | NM_021922 | 1256 | 357 | L>L |
| rs2075789 | 6 | MSH5 | Exon | 31708328 | NM_002441 | 371 | 29 | P>S |
| rs6907678 | 6 | FANCE | 5' near gene | 35418954 | NM_021922 | - | - | - |
| rs462779 | 6 | REV3L | Exon | 111695887 | NM_001286431 | 4364 | 1146 | T>I |
| rs9462088 | 6 | FANCE | Exon | 35430686 | NM_021922 | 1689 | 52 | A>T |
| rs316003 | 6 | SLC22A2 | Exon | 160645832 | NM_003058 | 168 | 52 | V>V |
| rs316019 | 6 | SLC22A2 | Exon | 160670282 | NM_003058 | 982 | 27 | S>A |
| rs1800629 | 6 | TNF | 5' near gene | 31543031 | NM_000594 | - | - | - |
| rs1883306 | 6 | POU3F2 | 5' near gene | 99279449 | NM_005604 | - | - | - |
| rs707937 | 6 | MSH5 | 3' near gene | 31731014 | NM_002441 | - | - | - |
| rs1869641 | 6 | POU3F2 | 5' near gene | 99277867 | NM_005604 | - | - | - |
| rs2444933 | 6 | POU3F2 | 5' near gene | 99278428 | NM_005604 | - | - | - |
| rs3823036 | 6 | POU3F2 | 3'-UTR | 99284532 | NM_005604 | 1953 | - | - |
| rs707939 | 6 | MSH5 | Intron | 31726688 | NM_002441 | - | - | - |
| rs1150793 | 6 | MSH5 | Intron | 31717696 | NM_002441 | - | - | - |
| rs2293852 | 6 | DDAH2 | 5' near gene | 31699980 | NM_013974 | - | - | - |
| rs3115672 | 6 | MSH5 | Exon | 31727897 | NM_002441 | 22 | 572 | T>T |
| rs409558 | 6 | MSH5 | Intron | 31708147 | NM_002441 | - | - | - |
| rs195862 | 6 | POU3F2 | 5' near gene | 99281809 | NM_005604 | - | - | - |
| rs3117572 | 6 | MSH5 | Intron | 31717692 | NM_002441 | - | - | - |
| rs707938 | 6 | SAPCD1 | 5' near gene | 31729359 | NM_001039651 | - | - | - |
| rs805304 | 6 | CLIC1 | 3' near gene | 31698088 | NM_001287593 | - | - | - |
| rs195854 | 6 | POU3F2 | 5' near gene | 99289475 | NM_005604 | - | - | - |
| rs1042544 | 6 | HLA_DPB1 | 3'-UTR | 33054457 | NM_002121 | 139 | - | - |
| rs117780937 | 6 | HLA_DQB1 | Intron | 32629685 | NM_001243961 | - | - | - |
| rs117988605 | 6 | HLA_DQB1 | Intron | 32628705 | NM_001243961 | - | - | - |
| rs149415995 | 6 | HLA_DRB5 | Exon | 32487205 | NM_002125 | 599 | 198 | E>E |
| rs28366217 | 6 | HLA_DRB1 | Intron | 32557328 | NM_001243965 | - | - | - |
| rs3204373 | 6 | HLA_DQB1 | Exon | 32632801 | NM_001243961 | 235 | 51 | N>N |
| rs3213487 | 6 | HLA_DQB2 | Intron | 32724317 | NM_001198858 | - | - | - |
| rs34236112 | 6 | HLA_DQB1 | 5'-UTR | 32634394 | NM_001243961 | 73 | - | - |
| rs34309628 | 6 | HLA_DRB1 | Intron | 32549331 | NM_001243965 | - | - | - |
| rs9272422 | 6 | HLA_DQA1 | 5' near gene | 32605132 | NM_002122 | - | - | - |
| rs9272742 | 6 | HLA_DQA1 | Intron | 32609722 | NM_002122 | - | - | - |
| rs9273528 | 6 | HLA_DQB1 | Intron | 32628633 | NM_001243961 | - | - | - |
| rs6915736 | 6 | SYNE1 | Intron | 152762098 | NM_033071 | - | - | - |
| rs9483504 | 6 | RPS12 | 5'-UTR | 133135886 | NM_001016 | 61 | - | - |
| rs1136633 | 6 | HLA_DRB5 | Exon | 32487170 | NM_002125 | 634 | 21 | T>M |
| rs144532965 | 6 | HLA_DRB5 | Exon | 32489795 | NM_002125 | 262 | 86 | D>V|G |
| rs28366215 | 6 | HLA_DRB1 | Intron | 32557310 | NM_001243965 | - | - | - |
| rs28366218 | 6 | HLA_DRB1 | Intron | 32557374 | NM_001243965 | - | - | - |
| rs28688207 | 6 | HLA_DQB1 | Intron | 32628660 | NM_001243961 | - | - | - |
| **Table S1.** (Continued) | | | | | | | | |
| rs11545686 | 6 | HLA_DQA1 | Exon | 32605288 | NM_002122 | 16 | 18 | M>T |
| rs7990 | 6 | HLA_DQA1 | Exon | 32609965 | NM_002122 | 61 | 183 | A>D |
| rs2294757 | 6 | VNN1 | Exon | 133035098 | NM_004666 | 97 | 26 | T>I |
| rs7385 | 6 | SENP6 | 3'-UTR | 76425594 | NM_001100409 | 4221 | - | - |
| rs7740756 | 6 | FUT9 | 3'-UTR | 96655105 | NM_006581 | 4415 | - | - |
| rs77866376 | 6 | HLA_DRB5 | Exon | 32487175 | NM_002125 | 629 | 28 | S>S |
| rs1799983 | 7 | NOS3 | Exon | 150696111 | NM_000603 | 119 | 298 | D>E |
| rs17420802 | 7 | PMS2 | Exon | 6017340 | NM_000535 | 2411 | 775 | N>S |
| rs1062372 | 7 | PMS2 | 5' near gene | 6048973 | NM_000535 | - | - | - |
| rs3213619 | 7 | ABCB1 | 5'-UTR | 87230193 | NM_000927 | 365 | - | - |
| rs6464268 | 7 | XRCC2 | 5' near gene | 152381150 | NM_005431 | - | - | - |
| rs2070744 | 7 | NOS3 | 5' near gene | 150690079 | NM_001160109 | - | - | - |
| rs2228006 | 7 | PMS2 | Exon | 6026775 | NM_000535 | 178 | 541 | K>E |
| rs1617640 | 7 | EPO | 5' near gene | 100317298 | NM_000799 | - | - | - |
| rs12536544 | 7 | RAC1 | Intron | 6435901 | NM_006908 | - | - | - |
| rs2868370 | 7 | HSPB1 | 5' near gene | 75930800 | NM_001540 | - | - | - |
| rs17064 | 7 | ABCB1 | 3'-UTR | 87133470 | NM_000927 | 4425 | - | - |
| rs10951983 | 7 | RAC1 | 3' near gene | 6446027 | NM_006908 | - | - | - |
| rs836548 | 7 | RAC1 | Intron | 6439448 | NM_006908 | - | - | - |
| rs3813517 | 7 | DAGLB | 3' near gene | 6448532 | NM_001142936 | - | - | - |
| rs4720672 | 7 | RAC1 | 3' near gene | 6443839 | NM_006908 | - | - | - |
| rs836554 | 7 | RAC1 | 3' near gene | 6445235 | NM_006908 | - | - | - |
| rs2009836 | 7 | HSPB1 | 5' near gene | 75931160 | NM_001540 | - | - | - |
| rs2070804 | 7 | HSPB1 | 3' near gene | 75933712 | NM_001540 | - | - | - |
| rs2868371 | 7 | HSPB1 | 5' near gene | 75930759 | NM_001540 | - | - | - |
| rs7459185 | 7 | HSPB1 | 3' near gene | 75934640 | NM_001540 | - | - | - |
| rs836556 | 7 | DAGLB | 3' near gene | 6447041 | NM_001142936 | - | - | - |
| rs2961047 | 7 | HSPB1 | 3' near gene | 75935056 | NM_001540 | - | - | - |
| rs2163938 | 7 | ABCA13 | Intron | 48415892 | NM_152701 | - | - | - |
| rs2280497 | 7 | ADCY1 | Intron | 45747933 | NM_021116 | - | - | - |
| rs2293106 | 7 | ADCY1 | Exon | 45753324 | NM_021116 | 318 | 13 | R>R |
| rs61128227 | 7 | CPED1 | Intron | 120773954 | NM_001105533 | - | - | - |
| rs143565372 | 7 | SEPT7P2 | ncRNA | 45736875 | [NC_000007](http://asia.ensembl.org/Homo_sapiens/Location/View?contigviewbottom=variation_feature_variation  0normal;db=core;source=dbSNP;v=rs143565372;vdb=variation;vf=28544203) | - | - | - |
| rs4236392 | 7 | FBXL18 | 3' near gene | 5487458 | NM_024963 | - | - | - |
| rs12666778 | 7 | PTPN12 | Intron | 77261820 | NM_001131008 | - | - | - |
| rs1045642 | 7 | ABCB1 | Exon | 87138645 | NM_000927 | 3928 | 1145 | I>I|I |
| rs2032582 | 7 | ABCB1 | Exon | 87160618 | NM_000927 | 317 | 893 | S>T|A |
| rs1805794 | 8 | NBN | Exon | 90990479 | NM_002485 | 663 | 185 | E>Q |
| rs2735383 | 8 | NBN | 3'-UTR | 90947269 | NM_002485 | 2916 | - | - |
| rs1801195 | 8 | WRN | Exon | 30999280 | NM_000553 | 41 | 174 | L>F |
| rs13312840 | 8 | NBN | 5' near gene | 90997909 | NM_002485 | - | - | - |
| rs2230009 | 8 | WRN | Exon | 30921935 | NM_000553 | 1128 | 114 | V>I |
| rs7760 | 8 | TP53INP1 | 3'-UTR | 95938422 | NM_001135733 | 543 | - | - |
| **Table S1.** (Continued) | | | | | | | | |
| rs28485205 | 8 | FABP9 | Intron | 82370696 | NM_001080526 | - | - | - |
| rs12674488 | 8 | MCPH1 | Exon | 6338306 | NM_024596 | 2121 | 682 | T>N |
| rs2929970 | 8 | WISP1 | 3'-UTR | 134241137 | NM_001204869 | 1939 | - | - |
| rs116716037 | 8 | WISP1 | 5'-UTR | 134203336 | NM_001204869 | 55 | - | - |
| rs2929973 | 8 | WISP1 | 3'-UTR | 134242508 | NM_001204869 | 331 | - | - |
| rs2013146 | 8 | WISP1 | 5' near gene | 134202992 | NM_001204869 | - | - | - |
| rs2929946 | 8 | WISP1 | Intron | 134220691 | NM_001204869 | - | - | - |
| rs2977529 | 8 | WISP1 | Intron | 134214906 | NM_001204869 | - | - | - |
| rs2977530 | 8 | WISP1 | Intron | 134215112 | NM_001204869 | - | - | - |
| rs4330674 | 8 | WISP1 | Intron | 134221502 | NM_001204869 | - | - | - |
| rs2977536 | 8 | WISP1 | Intron | 134219277 | NM_001204869 | - | - | - |
| rs16904853 | 8 | WISP1 | Intron | 134232303 | NM_001204869 | - | - | - |
| rs2929965 | 8 | WISP1 | Intron | 134235816 | NM_001204869 | - | - | - |
| rs2977519 | 8 | WISP1 | Intron | 134208839 | NM_001204869 | - | - | - |
| rs10956697 | 8 | WISP1 | Intron | 134230782 | NM_001204869 | - | - | - |
| RS3739261 | 8 | WISP1 | Exon | 134239770 | NM_001204869 | 572 | 156 | *>Q |
| rs3739262 | 8 | WISP1 | Intron | 134205713 | NM_001204869 | - | - | - |
| rs16893344 | 8 | WISP1 | Intron | 134206279 | NM_001204869 | - | - | - |
| rs10956696 | 8 | WISP1 | Intron | 134226879 | NM_001204869 | - | - | - |
| rs112897791 | 8 | WISP1 | 5' near gene | 134197982 | NM_001204870 | - | - | - |
| rs11778573 | 8 | WISP1 | Intron | 134228930 | NM_001204871 | - | - | - |
| rs138013484 | 8 | WISP1 | 3'-UTR | 134240098 | NM_001204872 | 9 | - | - |
| rs142461254 | 8 | WISP1 | 3'-UTR | 134240594 | NM_001204873 | 1396 | - | - |
| rs146162433 | 8 | WISP1 | 3'-UTR | 134241416 | NM_001204874 | 2218 | - | - |
| rs16904845 | 8 | WISP1 | 5' near gene | 134199776 | NM_001204875 | - | - | - |
| rs2013158 | 8 | WISP1 | 5' near gene | 134202942 | NM_001204876 | - | - | - |
| rs2929969 | 8 | WISP1 | 3'-UTR | 134240697 | NM_001204877 | 1499 | - | - |
| rs2929986 | 8 | WISP1 | Intron | 134211526 | NM_001204878 | - | - | - |
| rs2977537 | 8 | WISP1 | Intron | 134220063 | NM_001204879 | - | - | - |
| rs2977549 | 8 | WISP1 | 3'-UTR | 134242033 | NM_001204880 | 2835 | - | - |
| rs2977551 | 8 | WISP1 | 3'-UTR | 134243550 | NM_001204881 | 4352 | - | - |
| rs35472615 | 8 | WISP1 | 5' near gene | 134197537 | NM_001204882 | - | - | - |
| rs4265166 | 8 | WISP1 | 3'-UTR | 134241086 | NM_001204883 | 1888 | - | - |
| rs62514003 | 8 | WISP1 | 5' near gene | 134198196 | NM_001204884 | - | - | - |
| rs62514004 | 8 | WISP1 | 5' near gene | 134202489 | NM_001204885 | - | - | - |
| rs72731505 | 8 | WISP1 | 5' near gene | 134198960 | NM_001204886 | - | - | - |
| rs72731507 | 8 | WISP1 | 5' near gene | 134200038 | NM_001204887 | - | - | - |
| rs754958 | 8 | WISP1 | Intron | 134238771 | NM_001204888 | - | - | - |
| rs7828685 | 8 | WISP1 | Intron | 134224979 | NM_001204889 | - | - | - |
| rs7843546 | 8 | WISP1 | 5' near gene | 134198301 | NM_001204890 | - | - | - |
| rs4647554 | 9 | FANCC | 3'-UTR | 97862701 | NM_000136 | 3227 | - | - |
| rs1805329 | 9 | RAD23B | Exon | 110084328 | NM_001244713 | 825 | 228 | A>V |
| rs3176751 | 9 | XPA | 3'-UTR | 100437518 | NM_000380 | 1142 | - | - |
| **Table S1.** (Continued) | | | | | | | | |
| rs3176752 | 9 | XPA | 3'-UTR | 100437487 | NM_000380 | 1173 | - | - |
| rs10970975 | 9 | ACO1 | Intron | 32431931 | NM_001278352 | - | - | - |
| rs991121 | 9 | MEGF9 | Intron | 123370345 | NM_001080497 | - | - | - |
| rs12985 | 9 | ACO1 | 3'-UTR | 32450187 | NM_001278352 | 2975 | - | - |
| rs4836833 | 9 | PHF19 | 5' near gene | 123632829 | NM_001286842 | - | - | - |
| rs1800975 | 9 | XPA | 5'-UTR | 100459578 | NM_000380 | 114 | - | - |
| rs2228526 | 10 | ERCC6 | Exon | 50678717 | NM_000124 | 3465 | 197 | M>V |
| rs3740066 | 10 | ABCC2 | Exon | 101604207 | NM_000392 | 4219 | 1324 | I>I |
| rs6413432 | 10 | CYP2E1 | Intron | 135348544 | NM_000773 | - | - | - |
| rs2228527 | 10 | ERCC6 | Exon | 50678369 | NM_000124 | 3813 | 1213 | R>G |
| rs2228528 | 10 | ERCC6 | Exon | 50732280 | NM_000124 | 1372 | 399 | G>D |
| rs2273697 | 10 | ABCC2 | Exon | 101563815 | NM_000392 | 1496 | 417 | V>I |
| rs3793784 | 10 | ERCC6 | 5' near gene | 50747539 | NM_000124 | - | - | - |
| rs2031920 | 10 | CYP2E1 | 5' near gene | 135339845 | NM_000773 | - | - | - |
| rs717620 | 10 | ABCC2 | 5'-UTR | 101542578 | NM_000392 | 224 | - | - |
| rs1649942 | 10 | NRG3 | Intron | 83951691 | NM_001010848 | - | - | - |
| rs12220909 | 10 | MIR4293 | Exon | 14425221 | NR_036186 | - | - | - |
| rs3758391 | 10 | SIRT1 | 5' near gene | 69643342 | NM_001142498 | - | - | - |
| rs3740051 | 10 | SIRT1 | 5' near gene | 69643959 | NM_001142498 | - | - | - |
| rs4746720 | 10 | SIRT1 | 3'-UTR | 69676830 | NM_001142498 | 2271 | - | - |
| rs12778366 | 10 | SIRT1 | 5' near gene | 69643079 | NM_001142498 | - | - | - |
| rs1244229 | 10 | TAF3 | Exon | 8007560 | NM_031923 | 2293 | 696 | V>A |
| rs10787882 | 10 | - | Inter | 118936320 | - | - | - | - |
| rs10787889 | 10 | - | Inter | 118999311 | - | - | - | - |
| rs10886419 | 10 | LOC105378579 | Intron | 119204075 | - | - | - | - |
| rs11198727 | 10 | LOC105378579 | Intron | 119007585 | - | - | - | - |
| rs12571300 | 10 | LOC102724671 | - | 119004971 | - | - | - | - |
| rs17098456 | 10 | FAM45A | Intron | 119115290 | - | - | - | - |
| rs17668734 | 10 | SFXN4 | Intron | 119155180 | - | - | - | - |
| rs1998839 | 10 | - | Inter | 118912386 | - | - | - | - |
| rs2104529 | 10 | - | Inter | 118928746 | - | - | - | - |
| rs4751691 | 10 | - | Inter | 119090652 | - | - | - | - |
| rs549793 | 10 | - | Inter | 118978778 | - | - | - | - |
| rs690870 | 10 | - | Inter | 118994421 | - | - | - | - |
| rs9325562 | 10 | GRK5 | Intron | 119211031 | [NM_005308](https://www.ncbi.nlm.nih.gov/projects/sviewer/?id=NM_005308.2&search=NM_005308.2:c.52+3062G>A&v=1:100&content=5) | 3062 | - | - |
| rs10510053 | 10 | SFXN4 | Intron | 119148316 | [NM_213649](https://www.ncbi.nlm.nih.gov/projects/sviewer/?id=NM_213649.1&search=NM_213649.1:c.733-456G>A&v=1:100&content=5) | 733 | - | - |
| rs10787899 | 10 | EIF3A | Intron | 119039143 | [NM_003750](http://www.ncbi.nlm.nih.gov/projects/sviewer/?id=NM_003750.2&search=NM_003750.2:c.3527-704C>T&v=1:100&content=5) | 3527 | - | - |
| rs10886360 | 10 | - | Inter | 118994275 | - | - | - | - |
| rs11198678 | 10 | - | Inter | 118931127 | - | - | - | - |
| rs11198819 | 10 | - | Inter | 119201190 | - | - | - | - |
| rs12411538 | 10 | - | Inter | 118994784 | - | - | - | - |
| rs12412962 | 10 | SFXN4 | Intron | 119149801 | NM_213649 | - | - | - |
| rs12413092 | 10 | - | Inter | 118998581 | - | - | - | - |
| **Table S1.** (Continued) | | | | | | | | |
| rs17098212 | 10 | - | Inter | 118938043 | - | - | - | - |
| rs2275112 | 10 | SFXN4 | Intron | 119158104 | NM_213649 | - | - | - |
| rs2297696 | 10 | SFXN4 | Intron | 119156955 | NM_213649 | - | - | - |
| rs3802739 | 10 | FAM45A | Intron | 119129510 | NM_207009 | - | - | - |
| rs4336954 | 10 | GRK5 | Intron | 119217041 | NM_005308 | - | - | - |
| rs4752219 | 10 | - | Inter | 118856049 | - | - | - | - |
| rs4752263 | 10 | GRK5 | Intron | 119209705 | NM_005308 | - | - | - |
| rs4752269 | 10 | GRK5 | Intron | 119278440 | NM_005308 | - | - | - |
| rs683817 | 10 | - | Inter | 118979141 | - | - | - | - |
| rs7069995 | 10 | - | Inter | 118948655 | - | - | - | - |
| rs7072794 | 10 | - | Inter | 118991533 | - | - | - | - |
| rs7091672 | 10 | - | Inter | 118836909 | - | - | - | - |
| rs7092342 | 10 | - | Inter | 118893445 | - | - | - | - |
| rs7093673 | 10 | GRK5 | Intron | 119222844 | NM_005308 | - | - | - |
| rs7911636 | 10 | FAM45A | 5' near gene | 119103029 | NM_207009 | - | - | - |
| rs7914808 | 10 | GRK5 | Intron | 119241671 | NM_005308 | - | - | - |
| rs7920891 | 10 | FAM45A | Intron | 119135619 | NM_207009 | - | - | - |
| rs928670 | 10 | GRK5 | Intron | 119272147 | NM_005308 | - | - | - |
| rs10430633 | 10 | - | Inter | 118926084 | - | - | - | - |
| rs10787876 | 10 | - | Inter | 118896692 | - | - | - | - |
| rs10787945 | 10 | GRK5 | Intron | 119270615 | NM_005308 | - | - | - |
| rs10886342 | 10 | - | Inter | 118931320 | - | - | - | - |
| rs10886361 | 10 | LOC102724671 | - | 119005455 | - | - | - | - |
| rs10886406 | 10 | SFXN4 | Intron | 119162792 | NM_213649 | - | - | - |
| rs11198774 | 10 | FAM45A | 5' near gene | 119103720 | NM_207009 | - | - | - |
| rs11198793 | 10 | FAM45A | Intron | 119131072 | NM_207009 | - | - | - |
| rs12242686 | 10 | - | Inter | 118979937 | - | - | - | - |
| rs1409314 | 10 | LOC105378508 | Intron | 118856240 | - | - | - | - |
| rs1500986 | 10 | NANOS1 | 5' near gene | 119029035 | - | - | - | - |
| rs1511034 | 10 | FAM45A | Intron | 119105269 | - | - | - | - |
| rs1926581 | 10 | - | - | 118930334 | - | - | - | - |
| rs4237508 | 10 | - | - | 118911673 | - | - | - | - |
| rs4642993 | 10 | LOC105378508 | Intron | 118843830 | - | - | - | - |
| rs4752250 | 10 | FAM45A | Intron | 119116292 | NM_207009 | - | - | - |
| rs690987 | 10 | - | Inter | 119003615 | - | - | - | - |
| rs7079370 | 10 | - | Inter | 118885435 | - | - | - | - |
| rs7089521 | 10 | - | Inter | 119003280 | - | - | - | - |
| rs7905194 | 10 | - | Inter | 118938179 | - | - | - | - |
| rs7906373 | 10 | FAM45A | Intron | 119114555 | NM_207009 | - | - | - |
| rs915272 | 10 | FAM45A | Intron | 119133030 | NM_207009 | - | - | - |
| rs943269 | 10 | - | Inter | 118955606 | - | - | - | - |
| rs10510050 | 10 | LOC105378508 | 5' near gene | 118867050 | - | - | - | - |
| rs10787879 | 10 | - | Inter | 118928058 | - | - | - | - |
| **Table S1.** (Continued) | | | | | | | | |
| rs10886332 | 10 | - | Inter | 118891150 | - | - | - | - |
| rs10886341 | 10 | - | Inter | 118928275 | - | - | - | - |
| rs11198804 | 10 | SFXN4 | Intron | 119150273 | NM_213649 | - | - | - |
| rs11598851 | 10 | - | Inter | 118999058 | - | - | - | - |
| rs12775397 | 10 | - | Inter | 118999961 | - | - | - | - |
| rs2148496 | 10 | - | Inter | 118951825 | - | - | - | - |
| rs2271359 | 10 | FAM45A | Intron | 119105089 | NM_207009 | - | - | - |
| rs34179455 | 10 | FAM45A | 3'-UTR | 119137967 | NM_207009 | - | - | - |
| rs3858336 | 10 | FAM45A | Intron | 119118198 | NM_207009 | - | - | - |
| rs4751680 | 10 | - | Inter | 118889214 | - | - | - | - |
| rs4752217 | 10 | LOC105378508 | Intron | 118854484 | - | - | - | - |
| rs4752220 | 10 | LOC105378508 | Intron | 118856097 | - | - | - | - |
| rs7073467 | 10 | - | Inter | 119003294 | - | - | - | - |
| rs7089236 | 10 | - | Inter | 119003203 | - | - | - | - |
| rs7095989 | 10 | GRK5 | Intron | 119265585 | NM_005308 | - | - | - |
| rs7899785 | 10 | [LDHAP5](https://www.ncbi.nlm.nih.gov/gene/?term=729666%5BUID%5D) | Intron | 118933259 | - | - | - | - |
| rs7908018 | 10 | - | Inter | 118886308 | - | - | - | - |
| rs7923177 | 10 | LOC105378508 | Intron | 118847378 | - | - | - | - |
| rs189037 | 11 | ATM | 5'-UTR | 108093833 | NM_000051 | 275 | - | - |
| rs564250 | 11 | CAT | 5' near gene | 34458861 | NM_001752 | - | - | - |
| rs1001179 | 11 | CAT | 5' near gene | 34460231 | NM_001752 | - | - | - |
| rs3740615 | 11 | FANCF | 5'-UTR | 22647366 | NM_022725 | 22 | - | - |
| rs228589 | 11 | ATM | 5' near gene | 108093208 | NM_000051 | - | - | - |
| rs7943316 | 11 | CAT | 5'-UTR | 34460472 | NM_001752 | 1 | - | - |
| rs4585 | 11 | ATM | 3'-UTR | 108239628 | NM_000051 | 12949 | - | - |
| rs769217 | 11 | CAT | Exon | 34482908 | NM_001752 | 1256 | 389 | D>D |
| rs4442551 | 11 | FANCF | 5' near gene | 22648843 | NM_022725 | - | - | - |
| rs4447177 | 11 | FANCF | 3'-UTR | 22646025 | NM_022725 | 1363 | - | - |
| rs12806698 | 11 | RRM1 | 5'-UTR | 4115974 | NM_001033 | 51 | - | - |
| rs11020802 | 11 | MRE11A | 5' near gene | 94227125 | NM_005590 | - | - | - |
| rs10896607 | 11 | P2RX3 | Intron | 57106459 | NM_002559 | - | - | - |
| rs227091 | 11 | ATM | 3'-UTR | 108237839 | NM_000051 | 1116 | - | - |
| rs2659870 | 11 | DCHS1 | Intron | 6653232 | NM_003737 | - | - | - |
| rs2278900 | 11 | ZNF143 | Intron | 9495961 | NM_001282656 | - | - | - |
| rs12417980 | 11 | TRIM49 | Exon | 89531540 | NM_020358 | 1446 | 373 | G>R |
| rs649870 | 11 | DPAGT1 | Intron | 118971251 | NM_001382 | - | - | - |
| rs1695 | 11 | GSTP1 | Exon | 67352689 | NM_000852 | 562 | 15 | I>V |
| rs5744751 | 12 | POLE | Exon | 133253995 | NM_006231 | 964 | 252 | A>V |
| rs1051669 | 12 | WNK1 | 3' near gene | 1022452 | NM_001184985 | - | - | - |
| rs7963551 | 12 | WNK1 | 3' near gene | 1021515 | NM_001184985 | - | - | - |
| rs2234649 | 12 | TNFRSF1A | 5' near gene | 6451363 | NM_001065 | - | - | - |
| rs2279744 | 12 | MDM2 | 5' near gene | 69202580 | NM_001145337 | - | - | - |
| rs11614913 | 12 | HOXC10 | 3' near gene | 54385599 | NM_017409 | - | - | - |
| **Table S1.** (Continued) | | | | | | | | |
| rs10875989 | 12 | AQP2 | 3'-UTR | 50351075 | NM_000486 | 259 | - | - |
| rs296766 | 12 | AQP2 | 3'-UTR | 50350953 | NM_000486 | 2468 | - | - |
| rs3759125 | 12 | AQP2 | 5' near gene | 50343608 | NM_000486 | - | - | - |
| rs3759126 | 12 | AQP2 | 5' near gene | 50343862 | NM_000486 | - | - | - |
| rs461872 | 12 | LOC101927318 | 3' near gene | 50345206 | NR_110590 | - | - | - |
| rs7305534 | 12 | AQP2 | 5' near gene | 50341610 | NM_000486 | - | - | - |
| rs7314734 | 12 | AQP2 | 5' near gene | 50340041 | NM_000486 | - | - | - |
| rs63281060 | 12 | AEBP2 | intron | 19457018 | NM_153207 | - | - | - |
| rs7964052 | 12 | MDM1 | Intron | 68715417 | NM_001205028 | - | - | - |
| rs1801243 | 13 | ATP7B | Exon | 52548140 | NM_000053 | 1373 | 46 | S>A |
| rs1412125 | 13 | HMGB1 | 5' near gene | 31041595 | NM_002128 | - | - | - |
| rs2249825 | 13 | HMGB1 | Intron | 31037903 | NM_002128 | - | - | - |
| rs543304 | 13 | BRCA2 | Exon | 32912299 | NM_000059 | 434 | 1269 | V>V|V |
| rs206118 | 13 | BRCA2 | 5'-UTR | 32889792 | NM_000059 | 176 | - | - |
| rs1045411 | 13 | HMGB1 | 3'-UTR | 31033232 | NM_002128 | 373 | - | - |
| rs1061472 | 13 | ATP7B | Exon | 52524488 | NM_000053 | 2652 | 832 | K>R |
| rs9535826 | 13 | ATP7B | Intron | 52566126 | NM_000053 | - | - | - |
| rs9535828 | 13 | ATP7B | Intron | 52573422 | NM_000053 | - | - | - |
| rs873601 | 13 | ERCC5 | 3'-UTR | 103528337 | NM_000123 | 471 | - | - |
| rs7999812 | 13 | ATP7B | Intron | 52545495 | NM_000053 | - | - | - |
| rs1047768 | 13 | ERCC5 | Exon | 103504517 | NM_000123 | 564 | 46 | H>H |
| rs17655 | 13 | ERCC5 | Exon | 103528002 | NM_000123 | 3736 | 114 | D>H |
| rs175080 | 14 | MLH3 | Exon | 75513828 | NM_001040108 | 2747 | 844 | P>L |
| rs861539 | 14 | XRCC3 | Exon | 104165753 | NM_001100118 | 145 | 241 | T>M |
| rs2307486 | 14 | TMEM55B | 3' near gene | 20924204 | NM_001100814 | - | - | - |
| rs1130409 | 14 | TMEM55B | 3' near gene | 20925154 | NM_001100814 | - | - | - |
| rs1799794 | 14 | XRCC3 | 5'-UTR | 104179267 | NM_001100119 | 12 | - | - |
| rs2233406 | 14 | NFKBIA | 5' near gene | 35874799 | NM_020529 | - | - | - |
| rs11549465 | 14 | HIF1A | Exon | 62207557 | NM_001243084 | 245 | 66 | P>S |
| rs3212117 | 14 | XRCC3 | 3'-UTR | 104164687 | NM_001100118 | 1812 | - | - |
| rs3212118 | 14 | XRCC3 | 3'-UTR | 104164634 | NM_001100118 | 1865 | - | - |
| rs3212121 | 14 | XRCC3 | 3'-UTR | 104164522 | NM_001100118 | 1977 | - | - |
| rs34652473 | 14 | IGHV1-3 | 5' near gene | 106471595 | NC_000014 | - | - | - |
| rs1801321 | 15 | RAD51 | 5'-UTR | 40987565 | NM_001164270 | 239 | - | - |
| rs12593359 | 15 | RAD51 | 3'-UTR | 41023878 | NM_001164269 | 1669 | - | - |
| rs1801320 | 15 | RAD51 | 5'-UTR | 40987528 | NM_001164270 | 22 | - | - |
| [rs4417527](http://www.ncbi.nlm.nih.gov/projects/SNP/snp_ref.cgi?rs=4417527) | 15 | RAD51 | intron | 40729082 | [NM_001164269](http://www.ncbi.nlm.nih.gov/projects/sviewer/?id=NM_001164269.1&search=NM_001164269.1:c.647+258C>G&v=1:100&content=5) | - | - | - |
| rs28599926 | 15 | LOC101928039 | Intron | 22225320 | - | - | - | - |
| rs1516400 | 15 | AQP9 | 5' near gene | 58429264 | NM_020980 | - | - | - |
| rs1554203 | 15 | AQP9 | 5' near gene | 58429547 | NM_020980 | - | - | - |
| rs2077737 | 15 | AQP9 | 3' near gene | 58478950 | NM_020980 | - | - | - |
| rs8023369 | 15 | AQP9 | 3' near gene | 58482194 | NM_020980 | - | - | - |
| rs9920375 | 15 | AQP9 | 3' near gene | 58483073 | NM_020980 | - | - | - |
| **Table S1.** (Continued) | | | | | | | | |
| rs1867380 | 15 | AQP9 | Exon | 58476281 | NM_020980 | 1192 | 279 | T>A |
| rs3809581 | 15 | LOC102724099 | intron | 32620127 | XR_429506 | - | - | - |
| rs7496668 | 15 | ADAMTS17 | Exon | 100821576 | NM_139057 | 726 | 216 | S>L |
| rs1800566 | 16 | NQO1 | Exon | 69745145 | NM_000903 | 75 | 187 | P>S |
| rs11646374 | 16 | FANCA | Exon | 89857935 | NM_000135 | 1277 | 412 | A>V |
| rs1799801 | 16 | ERCC4 | Exon | 14041958 | NM_005236 | 2514 | 835 | S>S |
| rs2239359 | 16 | FANCA | Exon | 89849480 | NM_000135 | 1543 | 51 | G>S |
| rs9282681 | 16 | FANCA | Exon | 89805914 | NM_000135 | 424 | 1328 | T>A |
| rs7204252 | 16 | MVP | 3' near gene | 29864169 | NM_017458 | - | - | - |
| rs4788184 | 16 | MVP | 5' near gene | 29830426 | NM_005115 | - | - | - |
| rs4788186 | 16 | MVP | Intron | 29841225 | NM_005115 | - | - | - |
| rs1057451 | 16 | PAGR1 | 3'-UTR | 29833488 | NM_024516 | 3497 | - | - |
| rs11859599 | 16 | MPHOSPH6 | Intron | 82182832 | NM_005792 | - | - | - |
| rs2303262 | 16 | MPHOSPH6 | Exon | 82203758 | NM_005792 | 72 | 8 | R>K |
| rs3859104 | 16 | CES5A | Intron | 55895249 | NM_001143685 | - | - | - |
| rs9937572 | 16 | CES5A | Intron | 55890398 | NM_001143685 | - | - | - |
| rs4485435 | 17 | FASN | Exon | 80045086 | NM_004104 | 3384 | 189 | A>A |
| rs2243828 | 17 | MPO | 5' near gene | 56358884 | NM_000250 | - | - | - |
| rs2333227 | 17 | MPO | 5' near gene | 56358762 | NM_000250 | - | - | - |
| rs4246445 | 17 | FASN | Intron | 80039028 | NM_004104 | - | - | - |
| rs2048718 | 17 | INTS2 | 3' near gene | 59940819 | NM_020748 | - | - | - |
| rs2297518 | 17 | NOS2 | Exon | 26096597 | NM_000625 | 287 | 68 | S>L |
| rs4986765 | 17 | BRIP1 | Exon | 59763465 | NM_032043 | 2943 | 879 | E>E |
| rs11079454 | 17 | BRIP1 | 3'-UTR | 59757169 | NM_032043 | 7544 | - | - |
| rs799917 | 17 | BRCA1 | Exon | 41244936 | NM_007294 | 2844 | 871 | P>L|Q |
| rs1140616 | 17 | FASN | Exon | 80039481 | NM_004104 | 6519 | 2134 | I>I |
| rs1042522 | 17 | TP53 | Exon | 7579472 | NM_000546 | 417 | 72 | P>R |
| rs2228309 | 17 | FASN | Exon | 80051183 | NM_004104 | 684 | 189 | N>N |
| rs4986764 | 17 | BRIP1 | Exon | 59763347 | NM_032043 | 361 | 919 | S>P |
| rs2289669 | 17 | SLC47A1 | Intron | 19463343 | NM_018242 | - | - | - |
| rs28392491 | 17 | LGALS9C | 5' near gene | 18373249 | NM_001040078 | - | - | - |
| rs12727 | 17 | RPA1 | 3'-UTR | 1801065 | NM_002945 | 2562 | - | - |
| rs17339395 | 17 | RPA1 | 3'-UTR | 1802501 | NM_002945 | 3998 | - | - |
| rs9914073 | 17 | RPA1 | 3'-UTR | 1801592 | NM_002945 | 389 | - | - |
| rs17292622 | 17 | RPA1 | 3'-UTR | 1802418 | NM_002945 | 3915 | - | - |
| rs17339382 | 17 | RPA1 | 3'-UTR | 1801872 | NM_002945 | 3369 | - | - |
| rs3744768 | 17 | RPA1 | 3'-UTR | 1800886 | NM_002945 | 2383 | - | - |
| rs9082 | 17 | RPA1 | 3'-UTR | 1801263 | NM_002945 | 276 | - | - |
| rs3744766 | 17 | RPA1 | 3'-UTR | 1800949 | NM_002945 | 2446 | - | - |
| rs1131636 | 17 | RPA1 | 3'-UTR | 1801189 | NM_002945 | 2686 | - | - |
| rs5030740 | 17 | RPA1 | 3'-UTR | 1800600 | NM_002945 | 297 | - | - |
| rs3744767 | 17 | RPA1 | 3'-UTR | 1800932 | NM_002945 | 2429 | - | - |
| rs3744769 | 17 | RPA1 | 3'-UTR | 1800849 | NM_002945 | 2346 | - | - |
| **Table S1.** (Continued) | | | | | | | | |
| rs17734 | 17 | RPA1 | 3'-UTR | 1801144 | NM_002945 | 2641 | - | - |
| rs2302293 | 17 | ABCA9 | Intron | 66987202 | NM_080283 | - | - | - |
| rs3213690 | 17 | AMZ2 | Exon | 66246416 | NM_001033569 | 237 | 3 | N>D |
| rs736523 | 17 | MYO15B | Intron | 73588067 | NR_003587 | - | - | - |
| rs1052555 | 19 | ERCC2 | Exon | 45855524 | NM_000400 | 218 | 711 | D>D |
| rs1048290 | 19 | KEAP1 | Exon | 10600442 | NM_012289 | 1569 | 471 | L>L |
| rs11545829 | 19 | KEAP1 | Exon | 10599965 | NM_012289 | 1767 | 537 | Y>Y |
| rs25489 | 19 | XRCC1 | Exon | 44056412 | NM_006297 | 959 | 28 | R>H |
| rs238406 | 19 | ERCC2 | Exon | 45868309 | NM_000400 | 515 | 156 | R>R |
| rs2298881 | 19 | ERCC1 | 5' near gene | 45926916 | NM_202001 | - | - | - |
| rs12984195 | 19 | ERCC1 | 3'-UTR | 45410202 | NM_001983 | - | - | - |
| rs117128015 | 19 | PPP1R13L | 5' near gene | 45910903 | NM_001142502 | - | - | - |
| rs12983892 | 19 | CD3EAP | 3'-UTR | 45913309 | NM_012099 | 2571 | - | - |
| rs172731 | 19 | RAB3D | Intron | 11446951 | NM_004283 | - | - | - |
| rs7251786 | 19 | LPPR2 | 5' near gene | 11465129 | NM_001170635 | - | - | - |
| rs1046282 | 19 | PPP1R13L | 5' near gene | 45910672 | NM_001142502 | - | - | - |
| rs896412 | 19 | RAB3D | Intron | 11444131 | NM_004283 | - | - | - |
| rs2306190 | 19 | PALM3 | 3' near gene | 14162676 | NM_001145028 | - | - | - |
| rs2227270 | 19 | LSM4 | Intron | 18420704 | NM_001252129 | - | - | - |
| rs2903755 | 19 | ANKRD27 | Intron | 33098870 | NM_032139 | - | - | - |
| rs11615 | 19 | ERCC1 | Exon | 45923653 | NM_001166049 | 5 | 118 | N>N |
| rs13181 | 19 | ERCC2 | Exon | 45854919 | NM_000400 | 2298 | 751 | K>Q |
| rs1799793 | 19 | ERCC2 | Exon | 45867259 | NM_000400 | 981 | 312 | D>N |
| rs25487 | 19 | XRCC1 | Exon | 44055726 | NM_006297 | 1316 | 399 | Q>R |
| rs3212986 | 19 | ERCC1 | 3'-UTR | 45912736 | NM_001166049 | 1165 | - | - |
| rs4630 | 22 | GSTT1 | 3'-UTR | 24376322 | NM_000853 | 877 | - | - |
| rs2073774 | 22 | DGCR2 | Intron | 19028480 | NM_001173533 | - | - | - |
| rs2227291 | X | ATP7A | Exon | 77268502 | NM_000052 | 25 | 767 | V>L |
| rs6622665 | X | ATP7A | Intron | 77277268 | NM_000052 | - | - | - |
| rs17330644 | X | XIAP | 3'-UTR | 123043876 | NM_001167 | 4498 | - | - |
| rs28382752 | X | XIAP | 3'-UTR | 123045219 | NM_001167 | 5841 | - | - |
| rs28382740 | X | XIAP | 3'-UTR | 123041043 | NM_001167 | 1665 | - | - |
| rs28382751 | X | XIAP | 3'-UTR | 123045109 | NM_001167 | 5731 | - | - |
| rs28382746 | X | XIAP | 3'-UTR | 123910792 | NM_001167 | 3611 | - | - |

**Table S2A: Characteristics of subjects enrolled in discovery stage**

| **Characteristics** | **Response** | | **p** | **Overall toxicity** | | **p** | **Hematological toxicity** | | **p** | **Gastrointestinal toxicity** | | **p** |
| --- | --- | --- | --- | --- | --- | --- | --- | --- | --- | --- | --- | --- |
|  | Responders | Non-responders |  | Low toxicity | High toxicity |  | Low toxicity | High toxicity |  | Low toxicity | High toxicity |  |
| Total | 111 | 217 |  | 313 | 177 |  | 374 | 116 |  | 405 | 85 |  |
| Gender |  |  | 0.703 |  |  | 0.126 |  |  | 0.231 |  |  | 0.104 |
| Male | 83 | 158 |  | 239 | 124 |  | 282 | 81 |  | 306 | 57 |  |
| Female | 28 | 59 |  | 74 | 53 |  | 92 | 35 |  | 99 | 28 |  |
| Age(years) |  |  | 0.137 |  |  | 0.705 |  |  | 0.808 |  |  | 0.096 |
| <60 | 66 | 147 |  | 198 | 115 |  | 240 | 73 |  | 252 | 61 |  |
| ≥60 | 45 | 70 |  | 115 | 62 |  | 134 | 43 |  | 153 | 24 |  |
| Smoke condition |  |  | 0.741 |  |  | 0.635 |  |  | 0.713 |  |  | 0.918 |
| Smoker | 48 | 98 |  | 136 | 73 |  | 212 | 68 |  | 231 | 49 |  |
| Non-smoker | 63 | 119 |  | 177 | 104 |  | 162 | 48 |  | 174 | 36 |  |
| Histology |  |  | 0.000 |  |  | 0.009 |  |  | 0.002 |  |  | 0.851 |
| Adenocarcinoma | 39 | 130 |  | 176 | 74 |  | 207 | 43 |  | 206 | 44 |  |
| Squamous cell | 65 | 72 |  | 121 | 90 |  | 145 | 66 |  | 176 | 35 |  |
| Other | 7 | 15 |  | 16 | 13 |  | 22 | 7 |  | 23 | 6 |  |
| Pathological stage |  |  | 0.032 |  |  | 0.588 |  |  | 0.638 |  |  | 0.536 |
| III | 32 | 41 |  | 72 | 48 |  | 89 | 31 |  | 98 | 22 |  |
| IV | 74 | 170 |  | 225 | 121 |  | 264 | 82 |  | 289 | 57 |  |
| Other | 5 | 6 |  | 16 | 8 |  | 21 | 3 |  | 18 | 6 |  |
| PS |  |  |  |  |  |  |  |  |  |  |  |  |
| 1 | 4 | 9 | 0.941 | 13 | 10 | 0.408 | 18 | 5 | 0.759 | 18 | 5 | 0.437 |
| 2 | 93 | 200 |  | 262 | 141 |  | 304 | 99 |  | 340 | 63 |  |
| Other | 14 | 8 |  | 38 | 26 |  | 52 | 12 |  | 47 | 17 |  |

**Table S2B: Characteristics of subjects enrolled in validation stage**

| **Characteristics** | **Response** | | **p** | **Overall toxicity** | | **p** | **Hematological toxicity** | | **p** | **Gastrointestinal toxicity** | | **p** |
| --- | --- | --- | --- | --- | --- | --- | --- | --- | --- | --- | --- | --- |
|  | Responders | Non-responders |  | Low toxicity | High toxicity |  | Low toxicity | High toxicity |  | Low toxicity | High toxicity |  |
| Total | 151 | 630 |  | 573 | 215 |  | 607 | 175 |  | 722 | 66 |  |
| Gender |  |  | 0.388 |  |  | 0.029 |  |  | 0.355 |  |  | 0.152 |
| Male | 112 | 445 |  | 421 | 141 |  | 438 | 120 |  | 530 | 32 |  |
| Female | 39 | 185 |  | 152 | 74 |  | 169 | 55 |  | 192 | 34 |  |
| Age(years) |  |  | 0.071 |  |  | 0.533 |  |  | 0.325 |  |  | 0.775 |
| <60 | 75 | 364 |  | 326 | 117 |  | 348 | 93 |  | 407 | 36 |  |
| ≥60 | 76 | 266 |  | 247 | 98 |  | 259 | 82 |  | 315 | 30 |  |
| Smoke condition |  |  | 0.450 |  |  | 0.096 |  |  | 0.302 |  |  | 0.003 |
| Smoker | 59 | 268 |  | 230 | 101 |  | 249 | 80 |  | 292 | 39 |  |
| Non-smoker | 92 | 362 |  | 343 | 114 |  | 358 | 95 |  | 430 | 27 |  |
| Histology |  |  | 0.000 |  |  | 0.140 |  |  | 0.151 |  |  | 0.531 |
| Adenocarcinoma | 50 | 117 |  | 131 | 38 |  | 137 | 30 |  | 157 | 12 |  |
| Squamous cell | 67 | 426 |  | 357 | 141 |  | 379 | 115 |  | 455 | 43 |  |
| Other | 34 | 87 |  | 85 | 36 |  | 91 | 30 |  | 110 | 11 |  |
| Pathological stage |  |  | 0.213 |  |  | 0.049 |  |  | 0.066 |  |  | 0.211 |
| III | 47 | 181 |  | 177 | 54 |  | 186 | 44 |  | 216 | 15 |  |
| IV | 81 | 402 |  | 338 | 148 |  | 359 | 122 |  | 441 | 45 |  |
| Other | 23 | 47 |  | 58 | 13 |  | 62 | 9 |  | 65 | 6 |  |
| PS |  |  |  |  |  |  |  |  |  |  |  |  |
| 1 | 138 | 570 | 0.979 | 519 | 196 | 0.999 | 552 | 158 | 0.675 | 656 | 59 | 0.410 |
| 2 | 12 | 50 |  | 45 | 17 |  | 46 | 15 |  | 55 | 7 |  |
| Other | 1 | 10 |  | 9 | 2 |  | 9 | 2 |  | 11 | 0 |  |

**Table S3:** Paired gene-gene interaction in discovery stage and validation stage

| **Study Component** | **Phenotype** | **Locus1(chromatin)** | **SNP1** | **Gene1** | **Locus2(chromatin)** | **SNP2** | **Gene2** | **OR(95% CI)** | **P** |
| --- | --- | --- | --- | --- | --- | --- | --- | --- | --- |
|
| Discovery | Hematologic toxicity |  |  |  |  |  |  |  |  |
|  |  | 2 | rs17730989 | HSPD1 | 3 | rs2633851 | SUMF1 | 0.233 | 0.018 |
|  |  | 6 | rs28688207 | HLA-DQB1 | 17 | rs799917 | BRCA1 | 21.620 | 1.460×10^-5^ |
|  |  |  |  |  |  |  |  |  |  |
|  | Gastrointestinal toxicity |  |  |  |  |  |  |  |  |
|  |  | 6 | rs462779 | REV3L | 11 | rs189037 | ATM | 0.272 | 3.900×10^-5^ |
|  |  | 8 | rs2977530 | WISP1 | 10 | rs2228528 | ERCC6 | 6.780 | 7.220×10^-5^ |
|  |  | 6 | rs462779 | REV3L | 11 | rs228589 | NPAT | 0.306 | 9.370×10^-5^ |
|  |  |  |  |  |  |  |  |  |  |
|  | Overall toxicity |  |  |  |  |  |  |  |  |
|  |  | 4 | rs2231142 | ABCG2 | 16 | rs3859104 | CES5A | 8.044 | 4.350×10^-5^ |
|  |  | 2 | rs17730989 | HSPD1 | 3 | rs2633851 | SUMF1 | 0.084 | 4.670×10^-5^ |
|  |  | 5 | rs3776332 | ARHGAP26 | 7 | rs2070804 | HSPB1 | 6.376 | 5.310×10^-5^ |
|  |  |  |  |  |  |  |  |  |  |
|  | Sensitivity |  |  |  |  |  |  |  |  |
|  |  | 1 | rs4658 | SLC2A1 | 2 | rs17730989 | HSPD1 | 5.432 | 2.610×10^-5^ |
|  |  |  |  |  |  |  |  |  |  |
| Validation | Hematologic toxicity |  |  |  |  |  |  |  |  |
|  |  | 2 | rs17730989 | HSPD1 | 3 | rs2633851 | SUMF1 | 0.570 | 0.018 |
| **Table S3.** (Continued) | | | | | | | | | |
|  |  | 6 | rs28688207 | HLA-DQB1 | 17 | rs799917 | BRCA1 | 0.785 | 0.364 |
|  |  |  |  |  |  |  |  |  |  |
|  | Gastrointestinal toxicity |  |  |  |  |  |  |  |  |
|  |  | 6 | rs462779 | REV3L | 11 | rs189037 | ATM | 0.879 | 0.710 |
|  |  | 8 | rs2977530 | WISP1 | 10 | rs2228528 | ERCC6 | NA | NA |
|  |  | 6 | rs462779 | REV3L | 11 | rs228589 | NPAT | 1.744 | 0.116 |
|  |  |  |  |  |  |  |  |  |  |
|  | Overall toxicity |  |  |  |  |  |  |  |  |
|  |  | 4 | rs2231142 | ABCG2 | 16 | rs3859104 | CES5A | 1.871 | 0.012 |
|  |  | 2 | rs17730989 | HSPD1 | 3 | rs2633851 | SUMF1 | 0.713 | 0.120 |
|  |  | 5 | rs3776332 | ARHGAP26 | 7 | rs2070804 | HSPB1 | 1.446 | 0.066 |
|  |  |  |  |  |  |  |  |  |  |
|  | Sensitivity |  |  |  |  |  |  |  |  |
|  |  | 1 | rs4658 | SLC2A1 | 2 | rs17730989 | HSPD1 | 0.783 | 0.341 |
